# Genome-wide prediction of transcription factor binding sites using an integrated model

**DOI:** 10.1186/gb-2010-11-1-r7

**Published:** 2010-01-22

**Authors:** Kyoung-Jae Won, Bing Ren, Wei Wang

**Affiliations:** 1 University of California, San Diego, Department of Chemistry and Biochemistry, 9500 Gilman Drive, La Jolla CA 92093, USA; 2 Ludwig Institute for Cancer Research and Department of Cellular and Molecular Medicine, UCSD School of Medicine, 9500 Gilman Drive, La Jolla, CA 92093, USA

## Abstract

We present an integrated method called Chromia for the genome-wide identification of functional target loci of transcription factors. Designed to capture the characteristic patterns of transcription factor binding motif occurrences and the histone profiles associated with regulatory elements such as promoters and enhancers, Chromia significantly outperforms other methods in the identification of 13 transcription factor binding sites in mouse embryonic stem cells, evaluated by both binding (ChIP-seq) and functional (RNA interference knockdown) experiments.

## Abstract 2

A new approach for genome-wide transcription factor binding site prediction is presented that integrates sequence and chromatin modification data.

## Background

Transcription factors (TFs) play a central role in regulating gene expression. Binding of TFs to their target loci is a key step of activating or repressing a gene. Determination of transcription factor binding sites (TFBSs) is an important but challenging problem because the DNA segments recognized by TFs are often short and dispersed in the genome [1]. In addition, the target loci of a TF vary depending on tissue, stage of development or physiological condition. Such condition-dependent regulation makes the problem even more challenging.

Both experimental and computational technologies have been developed to identify TFBSs. Chromatin immunoprecipitation (ChIP)-chip [2,3] and, more recently, ChIP-seq have become popular and powerful tools to determine TFBSs at a genome-wide scale [3-5]. Currently, a major bottleneck in applying ChIP-chip or ChIP-seq to all TFs encoded in a genome is the availability of ChIP-quality antibodies against each TF. Efforts have been made to tag every individual TF but the success of tagging techniques has only been shown for a limited number of TFs in mammalian genomes.

Many computational methods [6-15] (for a survey, see [16]) have been developed to identify DNA segments recognized by TFs. These DNA motifs are often represented by a position-specific scoring matrix (PSSM) [17] that reflects the preference of nucleotides at each position. Because simply matching such DNA motifs in the genome always generates too many false positives, additional information, such as co-localization and conservation of TFBSs, are often included to improve prediction accuracy. Methods such as Comet [18], Cluster-Buster [19] and ModuleMiner [20] use motifs documented in databases - for example, JASPAR [21] and TRANSFAC [22] - or predicted by *de novo *motif finding algorithms, and search for clusters of TFBSs. Methods like Stubb [23] and EEL [24] also include motif conservation information in addition to TFBS clustering. Other methods such as CisModule [25] and EmcModule [26] conduct *de novo *motif finding and *cis*-regulatory module (CRM) identification simultaneously in an iterative fashion. Recently developed methods like GibbsModule [27] can further improve prediction accuracy by combining motif overrepresentation in the co-expressed genes, motif conservation and co-localization of TFBSs. Although all these methods showed promising performance on the test datasets, they are limited by various factors. For example, incorporation of conservation information can improve the prediction accuracy only if genomes with appropriate evolutionary distances are correctly selected and reliable alignment of these genomes, which is not a trivial task, can be generated. In addition, it is still challenging to apply many of these methods to predicting target loci of a TF at a genomic scale with acceptable accuracy. More importantly, none of these computational methods can work in a condition-dependent manner to distinguish TFBSs from one condition to another.

Recent mapping of histone modifications using ChIP-chip or ChIP-seq technologies [28,29] provides an opportunity of predicting TFBSs using an alternative approach. It has been shown that regulatory elements such as promoters and enhancers are associated with distinct chromatin signatures [28], and, conversely, such chromatin signatures could be used to predict the regulatory elements [28,30,31]. In the present study, we propose an integrated approach that combines sequence information and chromatin signatures to predict binding sites of individual TFs, rather than genomic regions of regulatory elements as in the previous studies. This method is called Chromia (CHROMatin based Integrated Approach). More specifically, we scored genomic sequences using a PSSM that represents the DNA motif recognized by a TF. The PSSM score pattern in a genomic region reflects the preference for binding of a TF. Such sequence information and ChIP-seq signals of histone modifications at promoters or enhancers were integrated using a hidden Markov model (HMM) that was designed to capture characteristic patterns of these signals. The HMM model was applied to genome-wide identifications of 13 TFBSs, including CTCF, E2F1, Esrrb, Klf4, c-Myc, n-Myc, Nanog, Oct4, Sox2, Smad1, STAT3, Tcfcp2l1, and Zfx, in mouse embryonic stem (mES) cells. The predictions were assessed using the ChIP-seq data of the same TFs [32], which showed that our approach outperformed many of the currently available methods in terms of both accuracy and efficiency.

## Results

### Chromatin signatures of promoters and enhancers

**Figure 1:**
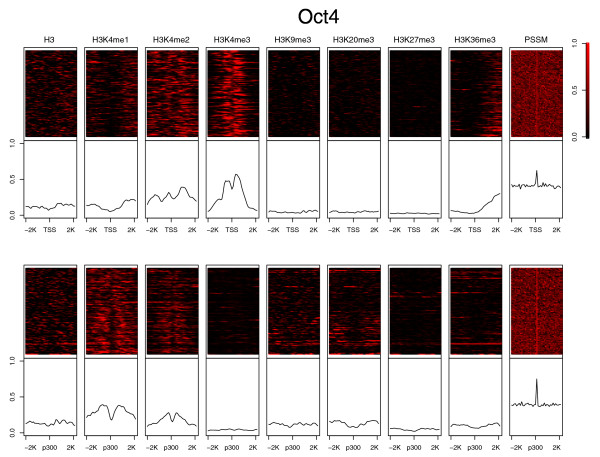
**Histone modification signals aligned at TSSs (promoters) and distal p300 binding sites (enhancers)**. Scaled signals of 100 promoter and enhancer regions with strong histone modification (sequencing read counts higher than an arbitrary cutoff) and the highest PSSM scores of Oct4 are plotted (see Materials and methods for how these regions were selected and Figure S1 in Additional file 1 for plots of other TFs). Individual histone marks are shown in a heatmap (upper panels) and the averaged signal is shown in the lower panels. TSSs are ordered by gene expression and the p300 binding sites are ordered using ChIP tag counts. Both are in descending order from the top to the bottom.

Distinct histone signatures have been observed at various genomic loci, including promoters and enhancers [28,29]. We first investigated the ChIP-Seq signals of eight chromatin marks (H3, H3K4me1, H3K4me2, H3K4me3, H3K9me3, H3K36me3, H3K20me3, and H3K27me3) aligned at transcription start sites (TSSs; promoters) in the mES cells [33] (Figure 1; Figure S1 in Additional file 1). The histone modification patterns at promoters are similar to what was previously observed. Namely, active marks, including mono-, di-, and tri-methylation of Lys4 of H3 (H3K4me1/2/3), showed strong signals; in contrast, the signals of repressive marks, such as H3K27me3, are much weaker. As the histone acetyltransferase p300 is commonly found at enhancer regions [34], we used the p300 binding sites located distal (>2.5 kb) from any RefSeq TSS [35] as a mark for enhancers. The eight histone marks at the p300 sites were not aligned as well as those at the promoters, which could be due to various reasons, such as different mES cell lines used in the p300 (E14 mouse ES cells) and histone modification ChIP-seq experiments (V6.5 cells) or noise in the p300 experiments. Nevertheless, chromatin marks at the enhancers still showed distinct patterns different from those at promoters - strong H3K4me1 and weak H3K4me3 signals, consistent with the previous observations [28,29] (Figure 1; Figure S1 in Additional file 1).

We also investigated the occurrences of the binding motifs of the 13 TFs in both promoters and enhancers (Figure 1; Figure S1 in Additional file 1). Peaks of PSSM scores were observed for all the TFs at both promoters and enhancers. The height of the peaks, which were affected by the alignment and/or enrichment of the TF binding motifs, varied for different TFs. Nanog, Oct4, Sox2 and Smad1 showed stronger PSSM score peaks at the enhancers than at the promoters (Table S1 in Additional file 2). In contrast, the other nine TFs showed better aligned and stronger peaks at the promoters than at the enhancers. Interestingly, CTCF, often serving as an insulator, is in the latter group, which may be due to its role of delineating alternative transcripts [36]. The alignment of histone marks and PSSM scores is consistent with the previous observation that Nanog, Oct4 and Sox2 tend to bind to enhancer regions [32]. Chen *et al*. [32] also suggested that both Smad1 and STAT3 binding sites were associated with Oct4-Sox2-Nanog-specific bindings sites. In contrast to Smad1, we observed that PSSM scores of STAT3 were much stronger in promoters than in enhancers, suggesting that STAT3 might not necessarily prefer binding to enhancers (also see below).

### Histone modification patterns aligned at TFBSs

**Figure 2:**
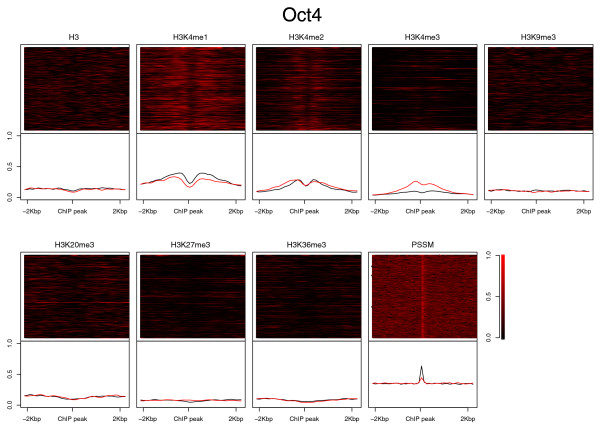
**Histone modification signals centered at the top 500 ChIP-seq binding peaks of Oct4 (heatmap in upper panels and the average shown as a black line in the lower panels)**. The red line in the lower panels is the averaged histone modification signals centered at the bottom 500 Oct4 binding peaks. The histone modification signals centered at other TF binding peaks are shown in Figure S2 in Additional file 1.

Given the binding data of the 13 TFs, we investigated whether any histone patterns are associated with a specific TF. We aligned the ChIP-seq signals of the eight histone marks centered at the top 500 binding peaks of the 13 TFs (Figure 2; Figure S2 in Additional file 1). We observed that the individual histone modifications at the TF binding sites varied significantly, but the average signal did show apparent patterns, particularly on H3K4me1/2/3: H3K4me1/2 presented a distinct bimodal profile in all TFBSs; H3K4me3 showed a strong peak in the binding sites of E2F1, c-Myc, n-Myc and Zfx, intermediate peaks for Esrrb, Klf4, STAT3 and Tcfcp2l1, and weak signals for CTCF, Nanog, Oct4, Smad1 and Sox2. H3K36me3 showed relatively strong signals for E2F1, c-Myc, n-Myc and Zfx (Figure S3 in Additional file 2). The repressive marks H3K9me3, H3K20me3 and H3K27me3 showed an overall low signal but individual sites fluctuate significantly.

Previous studies have shown distinct chromatin signatures of promoters and enhancers [28]: strong H3K4me1 and H3K4me3 in promoters compared to strong H3K4me1 and weak H3K4me3 in enhancers. The above analysis suggested the binding preferences of the TFs: E2F1, c-Myc, n-Myc and Zfx prefer promoters; Nanog, Oct4, Smad1 and Sox2 tend to bind to enhancers; and Esrrb, Klf4, Tcfcp2l1 and STAT3 have no preference. The genomic distributions of TF binding peaks determined in the ChIP-seq experiments indeed confirmed this prediction (Figure 2; Tables S1 and S2 in Additional file 2). Consistently, the binding peaks of c-Myc, n-Myc, Zfx, Klf4 and E2F1 contained a higher percentage of motifs in promoters than in enhancers(Table S1 in Additional file 2). Notably, of all the TF binding peaks in promoters, those of Oct4 contain the lowest percentage of its motif, which suggests many of the binding peaks in the promoters may result from indirect TF-promoter interactions. We also examined the TF binding peaks in the p300 binding regions that were distal to any annotated TSS (2,831 out of 3,684 p300 peaks) and presumably marked a portion of enhancers. The overlap between the binding peaks of the 13 TFs and p300 was smaller in enhancers, which was partially due to the incomplete representation of enhancers using the p300 binding sites. Nevertheless, the binding preference of the 13 TFs was consistent with the promoter analysis. Such binding location bias might also result in the observation that TFs other than Nanog, Oct4, Sox2, and Smad1 showed a higher percentage of motif occurrence in promoters than in enhancers (Table S1 in Additional file 2).

**Figure 3:**
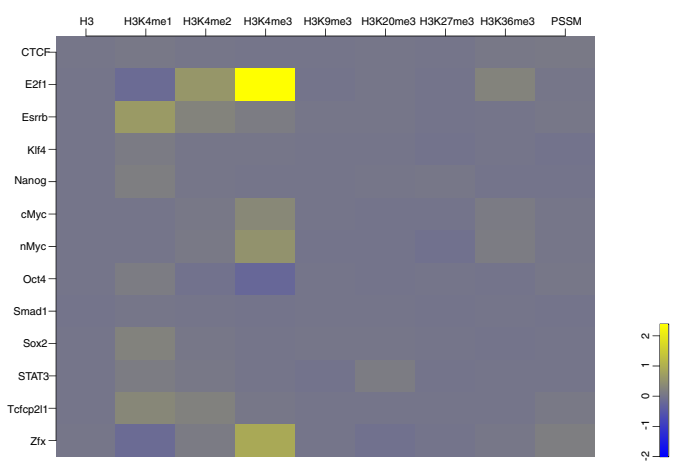
**Difference in histone mark signals associated with the binding strength of the 13 TFs**. We calculated the Euclidean distance between the averages of the histone mark signals (read count) centered at the top 500 and the bottom 500 TF binding peaks shown in the lower panels of Figure 2.

We next checked whether the binding strength of the TFs correlated with the histone modification patterns (Figure 2; Figure S2 in Additional file 1). We ranked the ChIP-seq peaks in each TF binding experiment based on the peak height. When examining the chromatin data for the top 500 and the bottom 500 TF binding peaks separately, we observed different signal strengths in histone marks (Figure 3). We also calculated the correlation of each histone mark and the TF binding strength using the top 500 and the bottom 500 binding peaks. (Table S3 in Additional file 2). We observed that, for example, the stronger the binding of E2F1 and c-Myc/n-Myc, the stronger the two promoter marks H3K4me3 and H3K36me3. Such a correlation was not unexpected and it might just reflect how preferable the TF binding sites were and/or how active the promoters were. The anti-correlation between Oct4 binding and H3K4me3 could also belong to this category because Oct4 preferred binding to enhancers and H3K4me3 usually showed weak or no signal at enhancers. We also observed that Zfx and E2f1, two promoter binders, were surprisingly anti-correlated with H3K4me1, although both were correlated with H3K4me2 and H3K4me3.

### Chromia: CHROMatin based Integrated Approach

**Figure 4:**
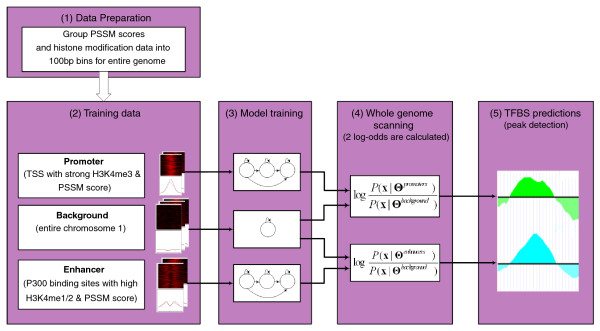
**The framework of Chromia**. (1) Data preparation. Chromia takes binned signals of PSSM scores and eight histone marks in the entire genome as input. (2) Training data. Regions centered at TSS and p300 binding sites were selected to train HMMs for promoters and enhancers, respectively. The entire chromosome 1 was used to train the background model. (3) Model training. Three HMMs with a left-right structure and a mixture of Gaussians were trained for promoters, enhancers and background, respectively. (4) Whole genome scanning. Two log-odd scores were calculated for each bin in the entire genome using the trained HMMs. (5) TFBS predictions. Log-odd scores of adjacent bins were averaged to smooth the curve. Bins with a log-odd score greater than other binding sites within ± 2,000 bp were predicted to contain the TFBSs. See Materials and methods for details.

Chromia integrates continuous (histone modifications) and discrete data (DNA sequence) in its model. It converts the discrete sequence data to continuous PSSM score signals. The binned histone modification and PSSM score are used as an input to the HMMs. Chromia uses three HMMs with a left-right structure and mixture of Gaussians to model promoter, enhancer, and background regions, respectively (Figure 4). RefSeq TSSs (promoter) and p300 binding sites (enhancer) with strong histone modification signals and PSSM scores for the TF(s) of interest (foreground) and the entire chromosome 1 (background) were selected to train the three HMMs, respectively. The trained HMMs were then used to identify genome-wide TFBSs. Using a sliding window, we calculated two log-odd scores (promoter against background and enhancer against background) for every bin in the entire genome. The peaks of the log-odd score were considered as putative TFBSs. The maximum log-odd peak was selected if multiple predictions were made within a given distance. As the histone sequencing reads were grouped to 100-bp bins, we were able to identify TF binding loci at a 100-bp resolution (see Materials and methods for details).

### Leave-one-chromosome-out cross-validation

The availability of the ChIP-seq experiments for the 13 TFs [32] in the mES cells provided an opportunity to assess the value of predicting TFBSs using chromatin signatures. We used the PSSM scores and the histone modification data aligned at TSS and p300 binding sites to train HMMs to capture characteristic patterns of these signals at promoters and enhancers. By scoring genomic loci using these HMMs (compared to a background HMM), we then made predictions of the binding sites of the TF(s) (see Materials and methods for details). We first evaluated the performance of Chromia using a leave-one-chromosome-out cross-validation, in which one chromosome was held out for testing and the remaining chromosomes were for training. This cross-validation was performed for all chromosomes. Based on the preference of binding to promoters or enhancers (see above analyses), we conducted this cross-validation on representative TFs, E2f1 for promoter predictions and the combined binding sites of Oct4, Sox2, and Nanog for enhancer predictions. For a fair comparison with other methods that required input of human-mouse sequence alignment, we removed ChIP-seq binding peaks residing in mouse genomic regions that were not aligned to the human genome in the UCSC genome browser. This way, 12,177 E2f1 and 16,377 Oct4-Sox2-Nanog ChIP-seq binding peaks were retrieved. A total of 90,000 regions with a length of 4,000 bp were randomly selected from the entire genome as negatives. Regions without alignment between human and mouse genomes and with weak histone modification signals (read count <10) were removed. As a result, 79,535 random regions in the entire genome were kept as negatives.

**Figure 5:**
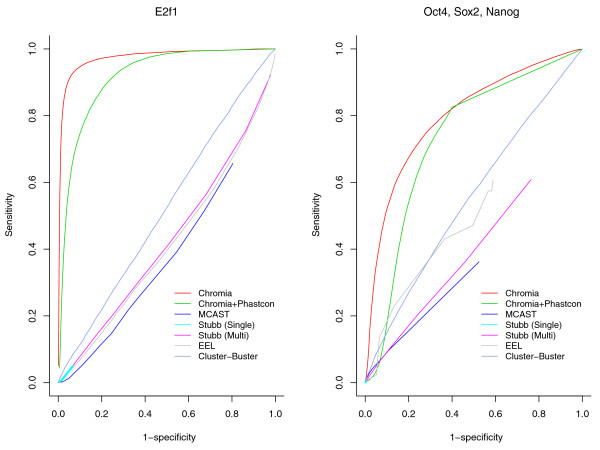
**ROC curves for TFBS identification methods in the leave-one-chromosome-out cross-validations**. Sensitivity = TP/(TP + FN) and Specificity = TN/(TN + FP).

We compared the performance of our method with several TFBS identification methods with publicly accessible software, including EEL [24], Cluster-Buster [19], Stubb [23] and MCAST [37]. Methods requiring inhibitive running time on the entire genome were not included in this comparison. Figure 5 shows the ROC curves for the leave-one-chromosome-out cross-validation using Chromia and several other computational methods. Table S4 in Additional file 2 compares the area under the receiver operator characteristic (ROC) curve (AUC) and the speed of all the tested methods. Obviously, Chromia outperformed all the other methods, demonstrating the effectiveness of our method. Interestingly, we observed that Chromia combined with Phastcon score [38] did not improve the performance.

**Figure 6:**
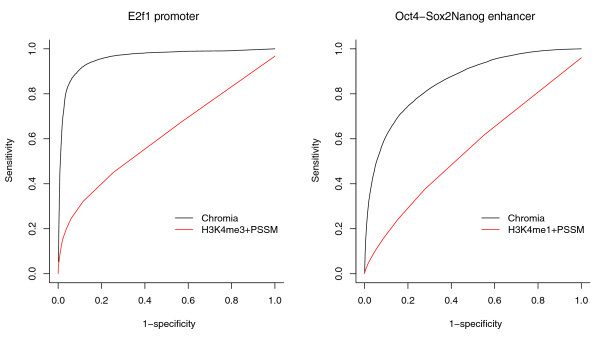
**ROC curves for TFBS identification using Chromia and the baseline method in the leave-one-chromosome-out cross-validation**. The baseline method used one histone mark read count (H3K3me3 for promoters and H3K4me1 for enhancers) multiplied by the PSSM score to rank all the sites.

Chromia uses HMMs to capture the special pattern of all eight histone marks and the PSSM scores in an integrated manner. To show the advantage of this approach, we also evaluated the performance of a baseline method that used the product of the PSSM score and a single histone mark read count (H3K4me3 for promoters and H3K4me1 for enhancer) to predict TFBSs. In order to make a rigorous comparison, we used binding sites of other TFs as negatives instead of using random sequences. Again, Chromia showed far better performance on all the TFs, except for CTCF, illustrating the advantage of using HMMs for TFBS prediction (Figure 6; Figures S4 and S5 and Table S5 in Additional file 2). For CTCF, neither Chromia nor the baseline method performed well, which is not unexpected as CTCF binding peaks do not match well with histone modification patterns (Figure 2; Figure S2 in Additional file 1). Overall, this comparison shows that using only one marker in the baseline method is not enough to build a good classifier. We also tried H3K4me2 alone or a combination of H3K4me1 and H3K4me2, and the performance of the baseline method did not change much. The superior performance of Chromia over the baseline method further emphasized the usefulness of an integrated model based on HMMs to capture spatial patterns of multiple chromatin marks.

### Genome-wide prediction of TFBSs using Chromia

In reality, what one cares about most is to predict TFBSs in the entire genome and reduce false positives among a given number of predictions. To evaluate how useful Chromia is to identify TFBSs at a genomic scale, we predicted the TFBSs for the 13 TFs in the mouse genome. We selected the top 2,000 predicted sites in promoters or enhancers and evaluated the prediction accuracy using the binding peaks of the TFs determined in the ChIP-seq experiments [32]. We considered a prediction as a true positive (TP) if there was a TF binding peak within a pre-defined distance |W| (we compare |W| = 1,000 bp in Table 1 and Additional file 2 (Table S6) and 500 bp in Additional file 2 (Table S7)); otherwise, the prediction was a false positive (FP). It is worth noting that the ChIP-seq experiments could be noisy even though we used them as the gold standard in accessing our predictions and the resolution of the binding sites is limited by the length of the DNA segments obtained in the ChIP-seq experiments, which is often around 500 bp. In addition, the TF binding peaks might be due to indirect interactions because no motif recognized by the TF could be found in many of these peaks.

**Table 1:** Assessment of the genome-wide TFBS predictions when |W| = 1,000 bp

	Chromia TP (PPV)	
	
TF	Promoters	Enhancers
CTCF	319 (16.0%)	195 (9.8%)
E2f1	1,920 (96.0%)	618 (30.9%)
Esrrb	585 (29.2%)	491 (24.6%)
Klf4	917 (45.9%)	351 (17.5%)
Nanog	138 (6.9%)	376 (18.8%)
Myc (n-Myc + c-Myc)	1,436 (71.8%)	167 (8.3%)
Oct4	240 (12.0%)	208 (10.4%)
Oct4-Sox2-Nanog	384 (19.2%)	431 (21.6%)
Smad1	6 (0.3%)	95 (4.8%)
Sox2	63 (3.1%)	235 (11.8%)
STAT3	99 (5.0%)	82 (4.1%)
Tcfcp2l1	716 (35.8%)	595 (29.8%)
Zfx	1,320 (66.0%)	219 (10.9%)

A prediction was considered to be a true positive (TP) if it was within |W| = 1,000 bp of a TF binding peak. The total number of predictions is 2,000.

We calculated positive predicative values (PPV = TP/(TP + FP)) of the predictions using various model configurations (Table 1; Table S6 in Additional file 2). E2f1, c-Myc, n-Myc and Zfx, which prefer promoters, achieved a PPV value greater than 60% for the promoter predictions. In contrast, the PPV values of enhancer predictions for these TFs were much worse. This observation is not surprising because these TFs tend to bind to promoters as shown above. When selecting the same number of predictions for both promoters and enhancers, the PPV for enhancers was expected to be lower than that for promoters. Another possible reason for low PPVs in enhancers was that the p300 binding sites only represented a portion of enhancers and the training histone data might not fully capture the chromatin signature associated with TFBSs in enhancers. Similarly, Nanog, Oct4, Smad1 and Sox2 prefer enhancers and showed higher PPVs in enhancers than in promoters. CTCF did not achieve a high PPV in either promoters (16.0%) or enhancers (9.8%), which might be due to lack of a definitive histone pattern associated with this insulator protein. Esrrb, Klf4, STAT3 and Tcfcp2l1 had comparable PPVs in promoters and enhancers, which is consistent with the above analyses that the binding sites of these TFs are a mixture of promoter and enhancer locations. As expected, there was a slight decrease of PPVs if we used a more stringent criteria of |W| = 500 bp (Table S7 in Additional file 2). We also observed that more predictions were made in promoters than in enhancers if using the same log-odd score cutoff (Table S8 and Figure S6 in Additional file 2), which suggested a better trained HMM for promoters.

The PPVs of Smad1 and STAT3 were low in both promoters and enhancers. The numbers of binding peaks of Smad1 and STAT3 determined by Chen *et al*. [32] were 1,126 and 2,546, respectively, which are much smaller than those of the other TFs (from 3,422 for n-Myc to 39,609 for CTCF). We suspect that the ChIP-seq peaks of these two TFs might be a result of indirect binding or noise in the experiments.

We then analyzed how sensitive our model is to the choice of PSSMs (Table S6 in Additional file 2). Instead of using the PSSMs found by MEME in the ChIP-seq binding peaks in the HMMs, we used the motifs documented in the TRANSFAC database [22] for Oct4 (access ID M01124), Sox2 (M01125), Nanog (M01123), Myc (M00055), E2f1 (M00939), Smad1 (M00701) and STAT3 (M00224). The motifs of the other TFs were not available in this database. Similar performance was observed for all but the E2f1 enhancer prediction.

Next, we investigated whether including conservation information (Phastcon score) [38] could improve the prediction accuracy (Table S6 in Additional file 2). Surprisingly, we found that including conservation in promoter predictions often deteriorated the performance. In contrast, conservation helped improve prediction accuracy in predicting TFBSs in enhancers for six TFs, including CTCF, E2f1, Klf4, c-Myc, n-Myc, and Zfx; however, none of these six TFs prefers binding to enhancers.

To further assess the performance of Chromia, we increased the number of predictions until FP = 2,000. We calculated AUC_2000 _values for the prediction of TFBSs in promoters and enhancers (Table S9 in Additional file 2). The AUC_2000 _values are significantly higher than those determined at random.

### Evaluating the genome-wide Chromia predictions using RNA interference experiments

RNA interference (RNAi) experiments in mES cells (E14/T21 cells) were conducted on 4 out of the 13 TFs to reveal genes potentially regulated by a specific TF [39]. We assessed our predictions using the 1,127, 1,365, 1,521 and 871 genes affected by knocking down Oct4, Sox2, Nanog and Esrrb, respectively [39]. To determine which genes were regulated by these TFBSs, we used a distance cutoff |W| = 2 kbp from the RefSeq TSSs for promoter predictions and |W| = 10 kbp for enhancer predictions. Namely, if a predicted TFBS using the promoter or enhancer HMM model was within 2 kbp (promoter prediction) or 10 kbp (enhancer prediction) of a RefSeq TSS, the gene was assumed to be regulated by the TF. In the TF binding experiments by Chen *et al*. [32], the number of binding peaks was in the range 3,761 (Oct4) to 2,1647 (Esrrb). Therefore, we made total predictions of 3,600, 8,000, 12,000 and 20,000, among which half were for promoters and half for enhancers, to compare with the TF binding experiments. A ChIP-seq binding peak was considered a TP if it is within 10 kbp of a RNAi-affected gene's TSS. Even when Chromia made less predictions than the number of TF binding peaks, we found our method still achieved better coverage, which is defined as correctly predicted genes among all genes affected by knocking down a specific TF (Table 2; Figure S7 in Additional file 2).

**Table 2:** Detection of genes affected by RNAi using genome-wide Chromia predictions and ChIP-seq binding peaks

		**Chen *et al***. [32]					
						
	Number of genes affected by RNAi	Number of TF binding peaks		Chromia TP total number (promoters and enhancers) of predictions^†^			
				
TFs			TP*	3,600	8,000	12,000	20,000
Nanog	1,521	10,343	265 (17.4%)	199 (13.1%)	**407 (26.8%)**	568 (37.3%)	843 (55.4%)
Oct4	1,127	3,761	151 (13.4%)	**170 (15.1%)**	327 (29.0%)	452 (40.1%)	652 (57.8%)
Sox2	1,365	4,526	137 (10.0%)	**195 (14.3%)**	372 (27.3%)	529 (38.8%)	753 (55.1%)
Esrrb	871	21,647	376 (43.2%)	143 (16.4%)	256 (29.4%)	349 (40.1%)	**476 (54.6%)**

*A TF binding peak in the Chen *et al*. study [32] was considered to be a TP if it was within |W| = 10 kbp of an RNAi-affected gene's TSS. PPV is shown in parenthesis.^†^The same number of predictions was made for promoters and enhancers. A promoter and an enhancer prediction were considered to be a TP if it was within |W| = 2 kbp for promoters and |W| = 10 kbp for enhancers of an RNAi-affected gene's TSS. The TP value from Chromia is shown in bold when it is larger than that from the Chen *et al*. study [32] but the number of Chromia predictions is smaller than the number of TF binding peaks determined in the Chen *et al*. study [32].

It is noteworthy that the RNAi, TF binding, and histone modification experiments were conducted in E14/T21, E14, and V6.5 mES cells, respectively. Even though the TF binding experiment was conducted in a cell line closer to the one used in the RNAi experiments, Chromia predictions based on the histone modification data obtained from a more distant cell line achieved better agreement with the knockdown assays. Recently, evidence has shown that enhancers are more cell type specific than promoters [40,41]. It is not surprising to observe more TPs in promoter predictions than in enhancer predictions. Nevertheless, our prediction identified a large portion of genes affected by RNAi experiments, demonstrating the usefulness of our approach to identify functional TFBSs at a genomic scale.

### Comparison with other methods

We compared the performance of our method with EEL [24], Cluster-Buster [19], Stubb [23] and MCAST [37]. We assessed the performance of all these methods using the TF binding peaks as the gold standard. We used the same distance cutoff |W| = 1,000 bp to decide whether a predicted TFBS was a TP. Because Stubb and EEL require pairwise alignment with other genomes and it was too time consuming to evaluate the performance of all methods using the entire genome, 20 chunks of genomic sequences (total 513,846,568 bp) that had pairwise alignment with the human genome were selected from the UCSC genome browser [42] for this comparison (Table S11 in Additional file 2).

Table 3 lists TPs and FPs of the predictions made by each method on each TF. We selected the top 600 predictions (combining promoter and enhancer predictions) with the largest log-odd score made by Chromia in these regions because the other methods did not have separate promoter and enhancer predictions. We found Chromia outperformed all the other methods for all TFs except CTCF. For CTCF, MCAST and Cluster-Buster performed the best, which might be due to the fact that the CTCF binding motif was very informative and/or only a small portion of CTCF binding sites were located in promoters (26.8%) or enhancers (0.9%) (Table S1 in Additional file 2).

**Table 3:** Comparison of several computational methods for predicting TFBSs in the 20 genomic regions of sequences

TF	Chromia	Cluster-Buster	EEL	MCAST	Stubb single	Stubb multiple
CTCF	79/521 (13.2%)	215/352 (37.9%)	20/19 (51.3%)	251/320 (44.0%)	24/155 (13.4%)	21/524 (3.9%)
E2f1	512/88 (85.3%)	7/550 (1.3%)	0/19 (0.0%)	3/578 (0.5%)	69/508 (12.0%)	48/509 (8.2%)
Esrrb	141/459 (23.5%)	28/542 (4.9%)	3/28 (9.7%)	94/486 (16.2%)	52/323 (13.9%)	27/504 (5.1%)
Klf4	205/395 (34.2%)	2/574 (0.3%)	2/33 (5.7%)	74/518 (12.5%)	165/412 (28.6%)	50/479 (9.5%)
Myc	347/253 (57.8%)	2/563 (0.4%)	3/39 (7.1%)	19/559 (3.3%)	76/301 (20.2%)	94/433 (17.8%)
Nanog	47/553 (7.8%)	2/554 (0.4%)	0/21 (0.0%)	4/571 (0.7%)	4/283 (1.4%)	1/550 (0.18%)
Oct4	90/510 (15.0%)	16/546 (2.8%)	0/44 (0.0%)	19/526 (3.5%)	1/192 (0.5%)	0/528 (0.0%)
Oct4-Sox2-Nanog	120/480 (120%)	22/541 (3.9%)	0/45 (0.0%)	8/551 (1.4%)	3/152 (1.9%)	6/501 (1.2%)
Smad1	6/594 (1.0%)	2/564 (0.4%)	0/33 (0.0%)	1/571 (0.2%)	0/188 (0.0%)	0/506 (0.0%)
Sox2	25/575 (4.2%)	14/560 (2.4%)	0/37 (0.0%)	16/551 (2.8%)	1/500 (0.2%)	1/120 (0.8%)
STAT3	6/594 (1.0%)	1/555 (0.2%)	0/34 (0.0%)	9/567 (1.6%)	3/99 (2.9%)	4/522 (0.8%)
Tcfcp2l1	203/397 (33.8%)	66/506 (11.5%)	2/38 (5.0%)	156/417 (27.2%)	10/69 (12.7%)	28/496 (5.3%)
Zfx	310/290 (51.7%)	1/560 (0.2%)	2/34 (5.6%)	146/443 (24.8%)	268/303 (46.9%)	140/398 (26.0%)

TP, FP and PPV (PPV = TP/(TP + FP)) are listed for each TF using every method.

**Figure 7:**
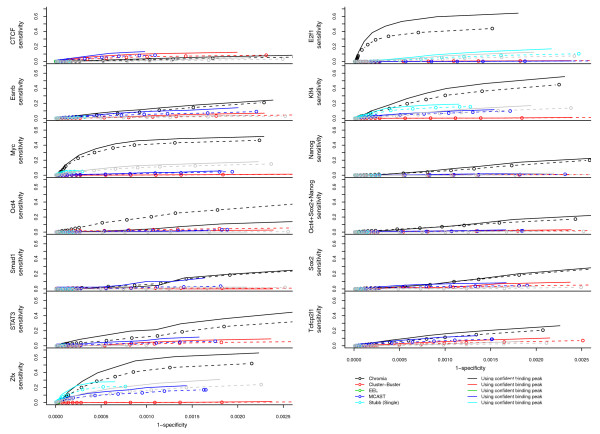
**ROC curves of TFBS identification using various methods for 20 genomic regions**. Sensitivity = TP/(TP + FN) and Specificity = TN/(TN + FP). Dotted lines are using all the ChIP-seq binding peaks and solid lines are using the confident ChIP-seq binding peaks that have strong motif scores in Table S1 in Additional file 2.

We also plotted ROC curves by changing the number of predictions made by each method (Figure 7). Because the number of true negatives (TNs) was very large, the specificity of all the listed methods was very high. Nevertheless, our method achieved higher AUC values (Table S10 in Additional file 2) than all other methods for all TFs but CTCF, which is consistent with the observation in Table 3.

The ChIP-seq experiments could be noisy and the binding peaks defined in these experiments could be due to indirect protein-DNA interactions. To obtain a set of highly confident binding peaks to assess the performance of each method, we selected the peaks that contained the binding motif recognized by the TF (the peaks listed in Table S1 in Additional file 2 using 500 bp as the distance cutoff). By changing the number of predictions made by our model, we plotted ROC curves in Figure 7. We observed better performance of our method using this evaluation set except Oct4.

## Discussion

The analysis of the recently available ChIP-seq data on 8 histone modification marks and 13 TF binding sites in mES cells confirmed the distinct chromatin signatures associated with promoters and enhancers. We did not observe any significant correlation between the histone modification patterns and the binding of the 13 TFs probably because none of these factors are involved in chromatin modification. The unexpected correlations between several histone marks and the binding strength of TFs (Table S3 in Additional file 2) still needs further validation and determination of the underlying molecular mechanisms.

Histone modifications reflect the epigenetic state of a cell, which provides useful information to map the functional activities of regulatory elements. In this study, we present a new computational model called Chromia that integrates sequence motif and chromatin signatures to predict target loci of TFs. We have demonstrated that the performance of our method is superior to many other methods. When comparing the predicted target genes of four TFs with the genes affected by knocking down these TFs, we found that Chromia identified more TF target genes than using the binding peaks of these TFs. This observation is not totally unexpected because the histone modifications are tightly related to function, which illustrates the usefulness of Chromia for predicting functional TFBSs.

There are several advantages of our approach. First, antibodies specifically against many histone marks are already available and therefore the chromatin modification profiles can be readily obtained for many organisms/tissues/cell lines. Second, this approach does not rely on the assumption that TFBSs are evolutionarily conserved, which allows identification of fast evolving or species-specific TFBSs. Furthermore, the non-trivial problem of choosing genomes with appropriate evolutionary distance and aligning these genomes can also be avoided. Third, since histone modification patterns are condition-specific, our method provides an approach to identifying TFBSs that may be functional only in specific tissues or developmental stages. Fourth, our method is much more efficient than many methods for predicting TFBSs at the genomic scale.

It is also worth noting that our model suggests a way to combine discrete and continuous sources of information by converting DNA sequence information to continuous PSSM scores. Previous studies showed that, in many scenarios, a cluster of weak TFBSs may play significant roles in regulating gene expression. The PSSM score profile provides an overall characterization of binding preference of a TF at a genomic locus. This is captured by the HMM and integrated with the chromatin signature to pinpoint the binding sites of a TF.

Recently, several approaches have been proposed to predict TFBSs in mammalian genomes using chromatin structure information. For example, ProbTB combined multiple sources of data to identify TFBSs in 47 mouse promoters [43]. Whitington *et al*. [44] used H3K4me3 as an additional filter to predict TFBSs in promoter regions. However, these studies are restricted to the small regions near TSSs. In contrast, we integrated chromatin signature and sequence motif information into one model and performed genome-wide prediction of TFBSs in both promoter and enhancer regions. Also, we demonstrated the superior performance of Chromia over the baseline method, which is in the same spirit of the Whitington *et al*. approach. Compared to our previous study [30], which aimed to find genomic regions of functional elements, including promoters and enhancers, here we were able to pinpoint TFBSs to 100-bp resolution by incorporating motif information, which also demonstrates the flexibility of our model to integrate additional data.

Although the performance of our method is very encouraging, it is no doubt there is still much room for improvement. Currently, only eight histone marks are mapped in the mES cells and not all of them are informative for locating regulatory elements. We expect that more histone marks with distinct patterns will help improve the performance of our method. We also observed that predictions for enhancers were relatively worse than those for promoters. Recent studies suggested that enhancers might be more cell type specific than promoters [40]. It is possible that the lower prediction accuracy for enhancers may be due to different cell lines used in histone modification (murine V6.5 ES cells) and TF binding (murine E14 ES cells) experiments. Furthermore, we should point out that our HMM was trained on the chromatin signatures associated with the p300 binding sites, which might only represent a small subset of the histone modification patterns at enhancers. Therefore, the trained HMM may miss many enhancers with different chromatin signatures. When binding sites of other cofactors commonly appearing at enhancers are mapped, a more comprehensive collection of histone modification patterns can be established and it is possible that the performance of our method can be further improved. Another limit of our method is that, like all methods that rely on binding motifs, it cannot distinguish TFs with very similar PSSMs (like n-Myc and c-Myc). However, if more histone marks are mapped and these TFs are associated with distinct chromatin signatures, it is possible to resolve the ambiguity of binding of these TFs.

Chromia is available at [45].

## Materials and methods

### Dataset

The histone modification data of eight chromatin marks in murine V6.5 ES cells were obtained from [33,35]. Based on our previous studies of smoothing ChIP-seq data, the sequencing reads were binned into 100-bp bins by averaging four adjacent 25-bp bins documented in [33,35]. PSSM scores were generated by a sliding window of the motif size, and the largest of the PSSM scores within a 100-bp bin was used as the value of that bin. These 100-bp binned values of sequencing read counts and PSSM scores were input to the HMM of Chromia.

### Data visualization: heatmaps

In plotting a heatmap of histone marks and the PSSM scores (but not in the HMM models), we re-scaled the signals in order to achieve a better visualization effect. Let **x **be the sequence read of a 100-bp bin. The re-scaled count in a bin *n*(**x**) is calculated as:

Where *x*_max _is the maximum sequencing read count of a histone mark in the entire genome, and *α*_*x *_is the value that only 0.1% of the histone mark bins have higher read counts. Each histone mark was re-scaled individually. In re-scaling the PSSM scores, *α*_*x *_was set to 15, which was the top 0.1% value of the PSSM scores in the entire genome. Plotting the re-scaled read counts avoided the problem that only the sites with large values (>0.1%) were visible with a bright color.

### Position specific scoring matrices (PSSMs) of the 13 TFs

Chen *et al*. [32] conducted ChIP-seq experiments on 13 TFs in the murine E14 ES cell. We extracted 200-bp sequences centered at each of the top 500 ChIP-seq binding sites of the 13 TFs. These sequences were input to the motif finding algorithm MEME [46]. We used the option '-dna -nmotifs 1 -mod oops -revcomp -minw 10 -maxw 15', which specified the number of motif, the oops assumption (one occurrence per sequence) and the range of the motif length (10 to 15 bp). By manually examining the motifs identified by MEME [46], all position specific frequency matrices (PSFMs) (Figure S8 and Table S1 in Additional file 2) were similar to those documented in the TRANSFAC database [22] and those reported in [32] by running the motif finding algorithms Weeder [47] or NestedMICA [48]. Because Oct4, Sox2 and Nanog are known to share many binding sites [49], we also searched for enriched motifs in the 1,500 peaks generated by pooling together the top 500 peaks of each TF. Such a 'combined' motif was similar to that found in a previous study [50].

A PSSM score was calculated for each motif:(1)

where *W*_*x *_is the width of the motif, *P*_*k*_(*x*_*k*_) and *P*^*b*^(*x*_*k*_) are the probabilities of observing nucleotide *x*_*k *_at position *k *from the motif and the background distributions, respectively. The background was obtained from the occurring frequency of each nucleotide in the entire mouse genome.

### The Chromia model

Chromia integrates continuous (histone modifications) and discrete data (DNA sequence) in its model. It converts the discrete sequence data to continuous PSSM score signals using Equation 1. As described above, the PSSM score was also binned to have the same format as the ChIP-seq data.

Chromia uses parallel HMM(***Θ***)s with a left-right structure [30,51] to integrate these two types of information. The left-right structure was chosen because it has been successfully applied to speech recognition, in which the speech signals are very similar to the ChIP-seq data and PSSM scores. In a left-right structure, transitions from state q to q'<q are not allowed. The first state has no transition from other states and the last state terminates the sequence. Transition is only allowed from state *i *to state *j *≥ *i*. In our previous study, this left-right structure has been successfully applied to capture the characteristic patterns of histone signatures [30]. The HMM has *Q *states. An HMM state emits a signal according to a probability density function of a Gaussian mixture of *N *dimensions. Here *N *is the total number of histone marks (*N*_*HistoneMark*_) and the PSSM score, that is, *N = N*_*HistoneMark *_+ 1. The probability density function of the Gaussian mixture is:

where ***x ***is the vector being modeled, *M *is the number of Gaussians and *c*_*jm *_is the mixture coefficient for the *m*th Gaussian distribution in state *j*; *G *[**x**, μ_*jm*_, U_*jm*_] represents the Gaussian function with a mean vector *μ*_*jm *_and a covariance matrix *U*_*jm*_. The forward and backward algorithm [51] was used to estimate the transition probabilities and the mixture coefficients as well as mean and covariance matrices of the Gaussians in each state. In this study, we chose to train three HMMs for promoters, enhancers and background separately. We set *Q *= 3 in the promoter and enhancer HMMs and *Q *= 1 in the background HMM. Strictly, it is a simple mixture of Gaussians when *Q *= 1. Each state was composed of three mixtures of Gaussian components (*M *= 3) to capture the complex signal patterns. Models with larger *M *did not improve the prediction performance (data not shown).

**Figure 8:**
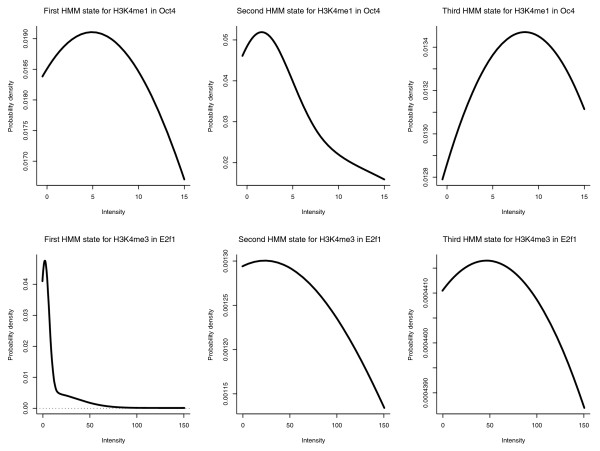
**Mixture of Gaussians in the trained HMM**. The x-axis is the signal intensity (sequencing read count) and the y-axis is the weighted sum of the three Gaussians in each state of the HMM. The first column shows the distribution of the mixture of Gaussians of the three states trained for H3K4me1 centered at the p300 binding sites (enhancers) with the strongest 100 Oct4 motif scores. Second column is for H3K3me3 in E2f1.

**Figure 9:**
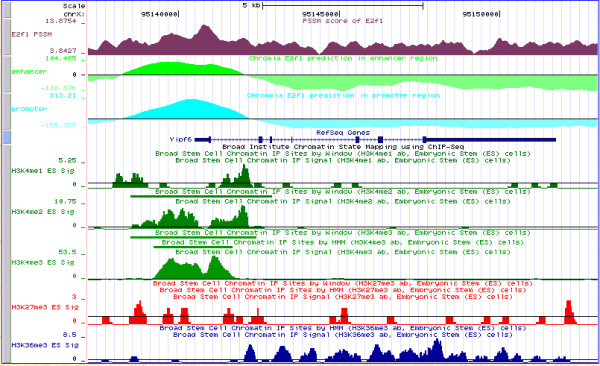
**HMM log-odds scores along with histone signatures and PSSM score around the TSS of gene *Yipf6***.

In our previous study [30], we investigated how to select *Q *to capture characteristic patterns of the histone modifications. We found the number of *Q *was related to the length of the genomic regions containing the histone modifications. Here, we considered 2,000-bp regions (20 100-bp bins) and *Q *was set to 3. This choice was particularly motivated by the observation of bimodal patterns for several histone marks, such as H3K4me1, in the promoters (Figure 1). Namely, the first and third state aimed to capture the two shoulder peaks and the second state the middle dip. In addition, mixtures of Gaussians allows modeling of the signal profiles better than individual Gaussians. In other words, the choice of *Q *= 3 and *M *= 3 is presumably better than *Q *= 9 and *M *= 1. We tested this in the leave-one-out validation using *Q *= 9, *M *= 1 and *Q *= 1, *M *= 3 (Figure S9 in Additional file 2). To further illustrate this point, we plotted the probability density versus sequencing read count in Figure 8 for the three states of the trained HMMs. The probability density of H3K4me1 trained with the histone marks centered at the p300 binding sites and with the strongest Oct4 PSSM motif scores has peaks of read count around 5, 3 and 9 for the first, second and third state of the HMM, correctly capturing the bimodal pattern of this mark. H3K4me3 is generally skewed towards TSS, which is consistent with the probability density peak at smaller read count in the first state and at larger ones in the next two states at the TSSs ranked highest with the E2f1 PSSM scores. An example, the promoter of *Yipf6*, is shown in Figure 9, in which the peaks of H3K4me3 and PSSM scores of E2f1 are located downstream of the TSS; Chromia correctly predicted this region as a promoter. It is worth pointing out that chromatin profiles at individual sites are not necessarily aligned well to the average pattern (Figure 1; Figure S1 in Additional file 2). We observed that a single Gaussian distribution was often not able to model the individual profiles as well as a mixture of three (or more) Gaussians.

### Training set

To train an HMM that integrated chromatin signatures and the motif information, we selected regions containing both strong histone modification signals and large PSSM scores because the HMM model was designed to capture patterns of chromatin and sequence motif data. To select strong histone modification signals, we first chose a read count cutoff where only 1% of all bins in all chromosomes had a value larger than the cutoff. We selected H3K4me3 (cutoff = 13.6) as the mark for promoters (annotated RefSeq TSSs) and H3K4me1 (cutoff = 5.9) or H3K4me2 (cutoff = 7.6) as the marks of enhancers (p300 binding sites). The prediction results were not sensitive to the cutoff value (data not shown). Next, all the selected promoter or enhancer bins in the entire genome that contain a sequencing read count larger than the cutoff value were ranked using the PSSM score of the TF under consideration. The top 100 promoters and 100 enhancers were then selected as the training set (Figure 1; Figure S1 in Additional file 1). The background model was trained on the entire chromosome 1.

### Genome-wide predictions of the TFBSs using the Chromia model

For a given genomic region, likelihood scores were calculated using the three HMMs for promoter, enhancer and background separately. The likelihood of an input ***x ***(chromatin and sequence data) was calculated by summing over all possible paths through the hidden states.

where ***q ***is the state of the HMM(Θ) (promoter, enhancer or background). In Chromia, two log-odd scores, one for promoter and one for enhancer predictions, were calculated as:(2)

The log-odd score reflects how strong a signal is compared to the background and has been widely applied, for example, to calculating the conservation score in phylo-HMM [38].

To incorporate conservation information, we further multiplied the Phastcon score [38]. The maximum Phastcon score in a bin was used as *Score*_*Phastcon *_in the following equations (Table S6 in Additional file 2):(4)

We calculated the log-odd scores for both promoters and enhancers using a sliding window of 2,000 bp centered at each bin. We smoothed the results by averaging the scores of the three adjacent bins. Among log-odds for promoters and enhancers, we only considered bins as potential TFBS-containing regions if they had a log-odd score larger than all other bins within ± 2,000 bp. We kept all the potential TFBS-containing bins if the distance between them was greater than 2,000 bp.

### Running other programs

All programs were run using their default setup and parameters. To run MAST we used the background obtained by running MEME [46]. Especially, for cross-validation, we tested MAST [52] on the sequences whose alignment between human and mouse genomes was available in the UCSC genome browser. We used the option '-comp' to select the current target sequences as a random model and '-ev 1000000' to obtain output with various E-values. We used different cutoffs for E-value to draw ROC curves. MCAST was run with an option '-e-thresh 0' to turn off thresholding. We changed the motif score to draw ROC curves. Cluster-Buster [19] was run with an option '-p0 -m0 -c0' to get the output not using pseudocounts (because pseudocounts were already included in the PSSM) and without thresholding the motif and cluster scores. We used a cluster score threshold as a cutoff to draw ROC curves. To run EEL [24] and Stubb [23], we used human and mouse orthologous sequences obtained from the UCSC genome browser. EEL aligned the orthorlogous sequences and yielded a binding score, which was changed to plot ROC curves. To run Stubb using its multiple sequence option, we used LAGAN [53] to align human and mouse orthorlogous sequences and used 'window size' = 500 and 'shiftsize' = 100. We changed the free energy calculated by Stubb to plot ROC curves.

### Calculating AUC_2000 _of Chromia and plotting ROC curves for method comparisons

To evaluate the performance of the genome-wide TFBS predictions made by Chromia, we calculated the AUC when FP = 2000 (AUC_2000_; Table S9 in Additional file 2). For comparison between different methods using the 20 large chunks of genomic regions, we plotted ROC curves (Figure 7). In both of the above situations, we scored every 100-bp bin in large genomic regions and the number of TNs was huge. To make it possible to draw a ROC curve and calculate the AUC, we grouped the adjacent ten 100-bp bins into one 1,000-bp bin. This 1,000-bp bin was considered a TP if it contained a ChIP-seq binding peak and was predicted to contain a TFBS; otherwise, it was a FP. A TN was a 1,000-bp bin that did not contain any ChIP-seq binding peak and was not predicted to contain a TFBS; otherwise, it was a false negative (FN).

## Abbreviations

AUC: area under the ROC curve; ChIP: chromatin immunoprecipitation; FN: false negative; FP: false positive; HMM: hidden Markov model; mES: mouse embryonic stem; PPV: positive predicative value; PSSM: position specific scoring matrix; RNAi: RNA interference; ROC: receiver operator characteristic; TF: transcription factor; TFBS: transcription factor binding site; TN: true negative; TP: true positive; TSS: transcription start site.

## Authors' contributions

KJW and WW conceived and designed the experiments. KJW analyzed the data. WW and BR contributed to analysis of the data. KJW and WW wrote the manuscript. All authors read and approved the final manuscript.

## Supplementary Material

Additional file 1 Figures S1 and S2 Click here for file

Additional file 2 Supplementary text, Figures S3 to S10, and Tables S1 to S11 Click here for file
