# Supplementary figures and images for: Genome-wide prediction of transcription factor binding sites using an integrated model
Source: Genome Biol. 2010 Jan 22;11(1):R7. doi: 10.1186/gb-2010-11-1-r7 (PMC2847719; doi:10.1186/gb-2010-11-1-r7)

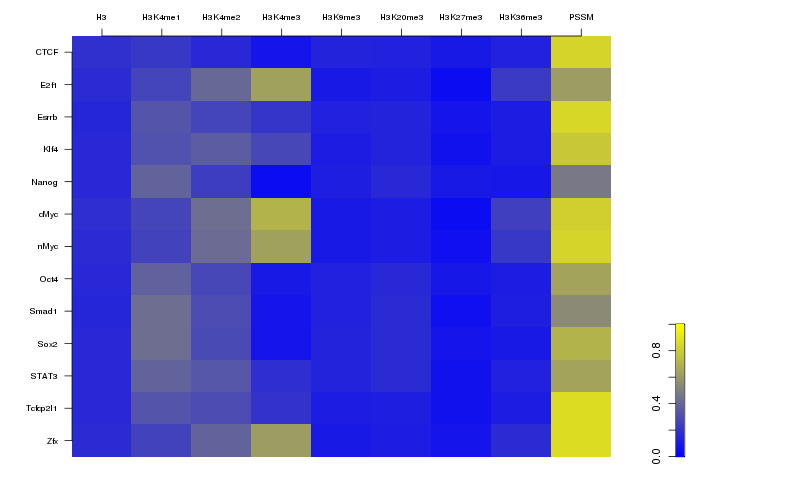

Supplement: Additional file 1 — Figures S1 and S2 [file gb-2010-11-1-r7-S1.zip › FigS3HistoneIntensity.png]

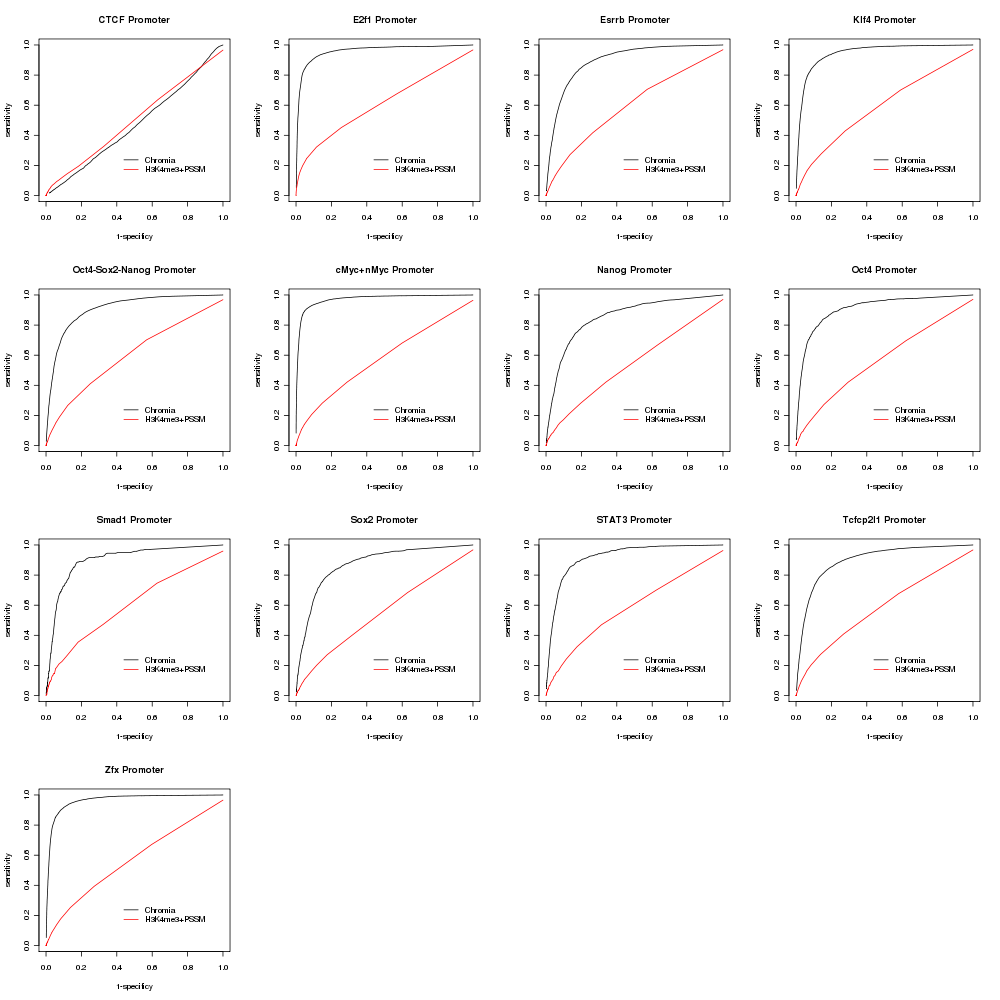

Supplement: Additional file 1 — Figures S1 and S2 [file gb-2010-11-1-r7-S1.zip › FigS4.Base.pro2.png]

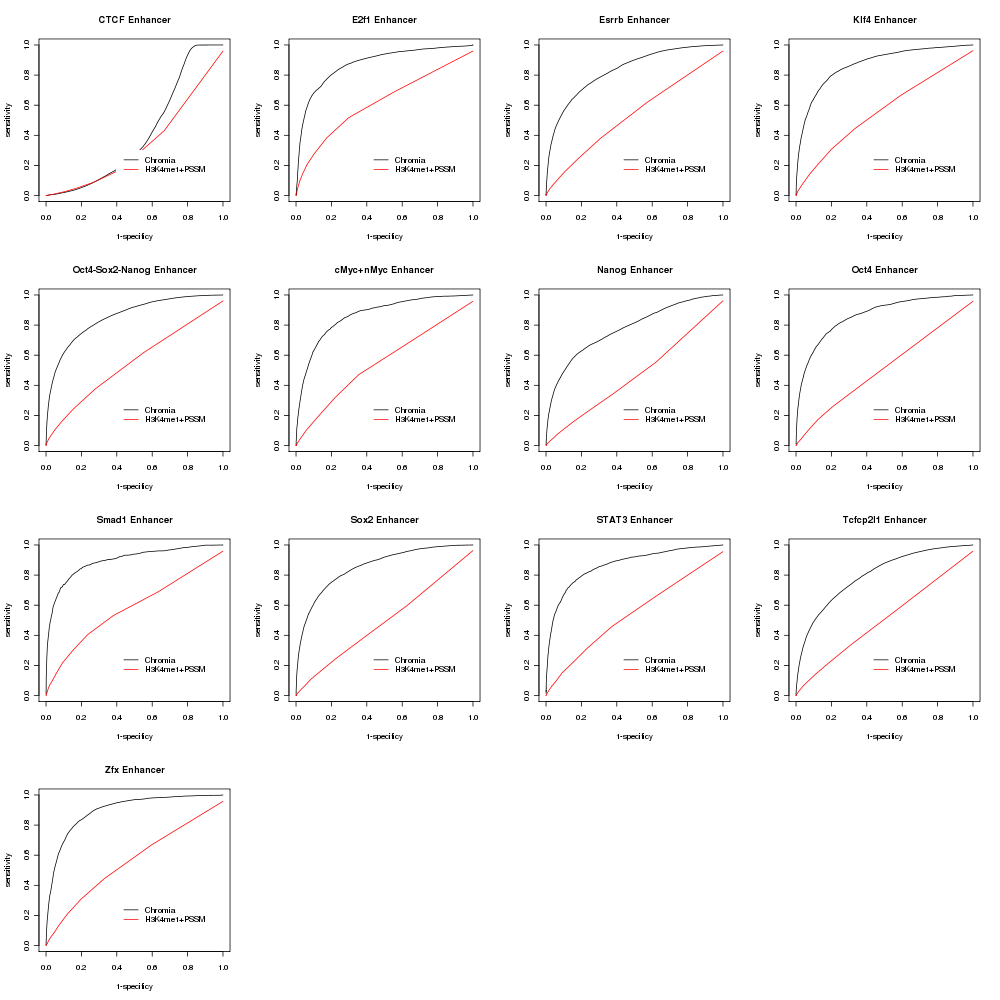

Supplement: Additional file 1 — Figures S1 and S2 [file gb-2010-11-1-r7-S1.zip › FigS5.Base.enh2.png]

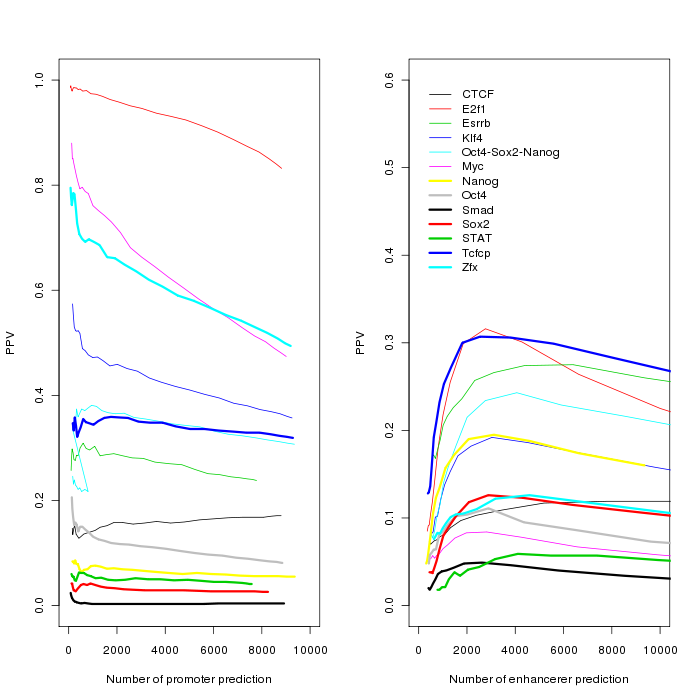

Supplement: Additional file 1 — Figures S1 and S2 [file gb-2010-11-1-r7-S1.zip › FigS6.ProEnh.png]

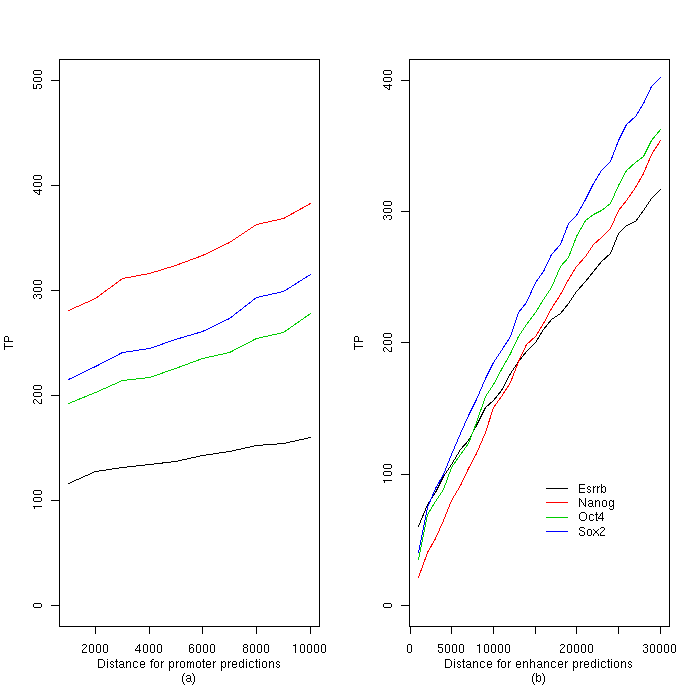

Supplement: Additional file 1 — Figures S1 and S2 [file gb-2010-11-1-r7-S1.zip › FigS7.rnai.png]

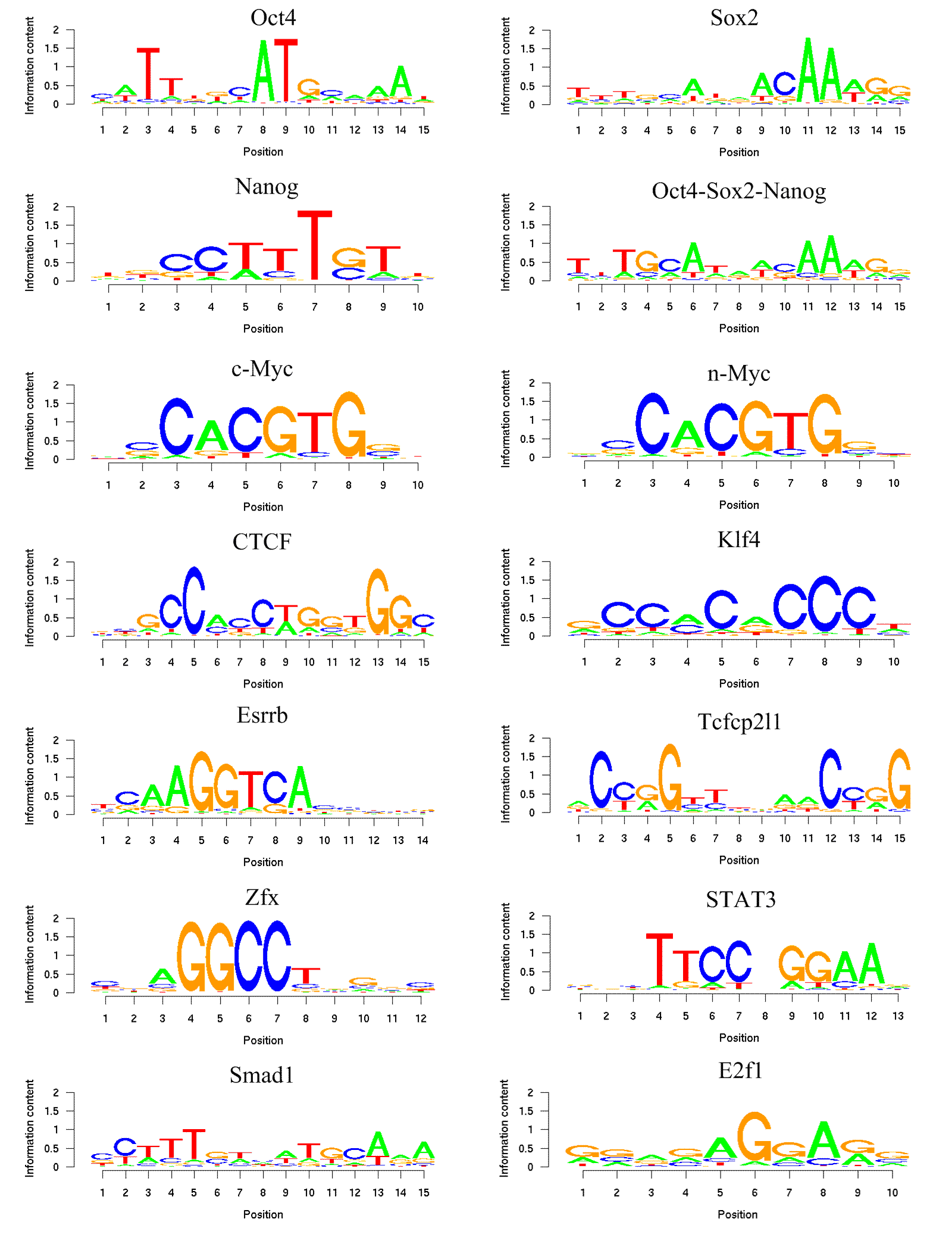

Supplement: Additional file 1 — Figures S1 and S2 [file gb-2010-11-1-r7-S1.zip › FigS8.PSSM.gif]

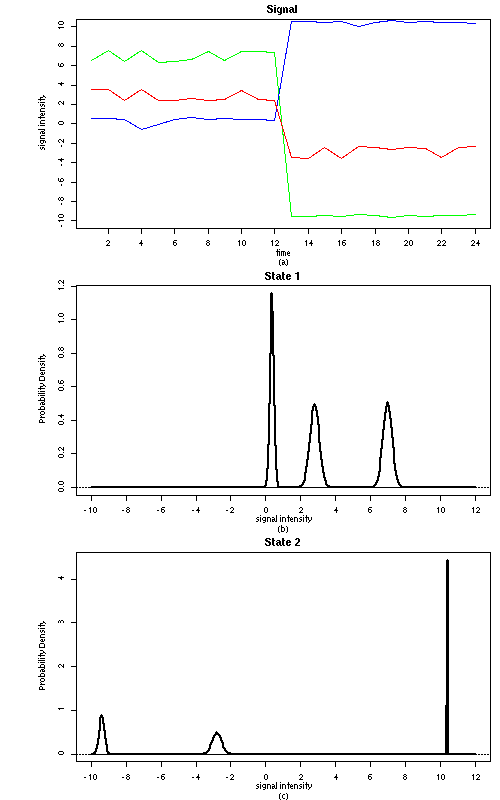

Supplement: Additional file 1 — Figures S1 and S2 [file gb-2010-11-1-r7-S1.zip › FigS9.png]

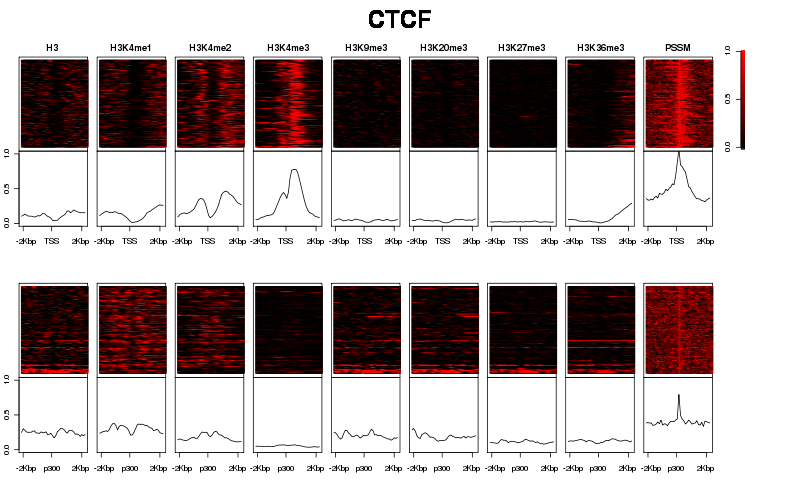

Supplement: Additional file 1 — Figures S1 and S2 [file gb-2010-11-1-r7-S1.zip › S1.CTCF.png]

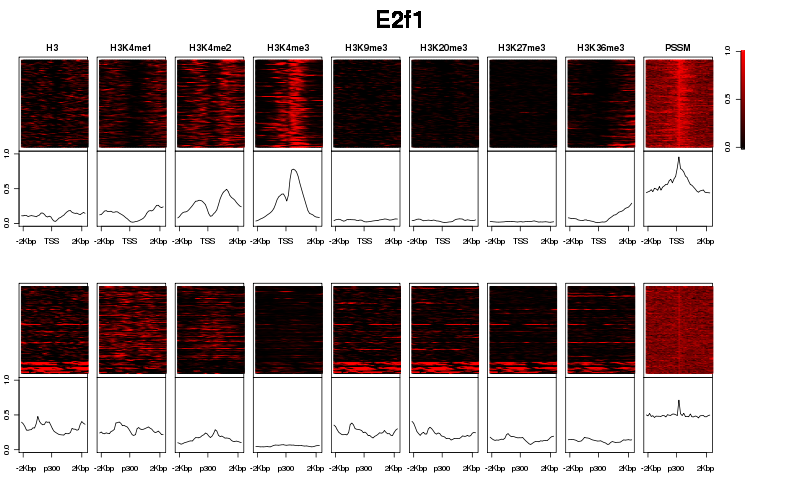

Supplement: Additional file 1 — Figures S1 and S2 [file gb-2010-11-1-r7-S1.zip › S1.E2f1.png]

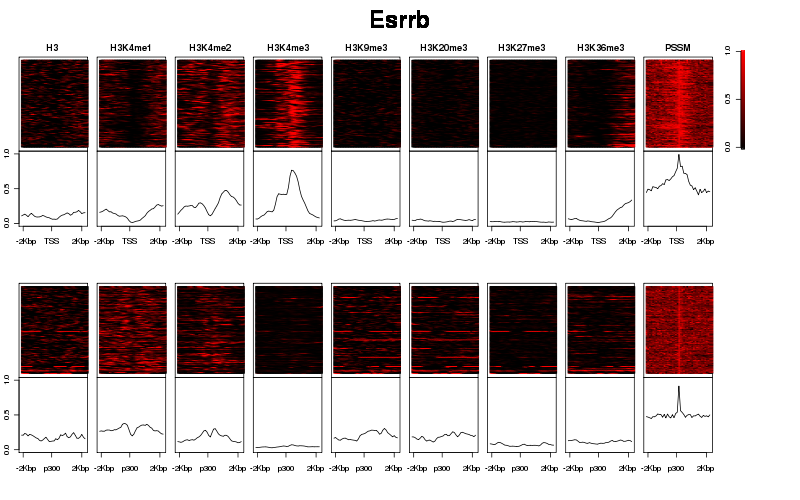

Supplement: Additional file 1 — Figures S1 and S2 [file gb-2010-11-1-r7-S1.zip › S1.Esrrb.png]

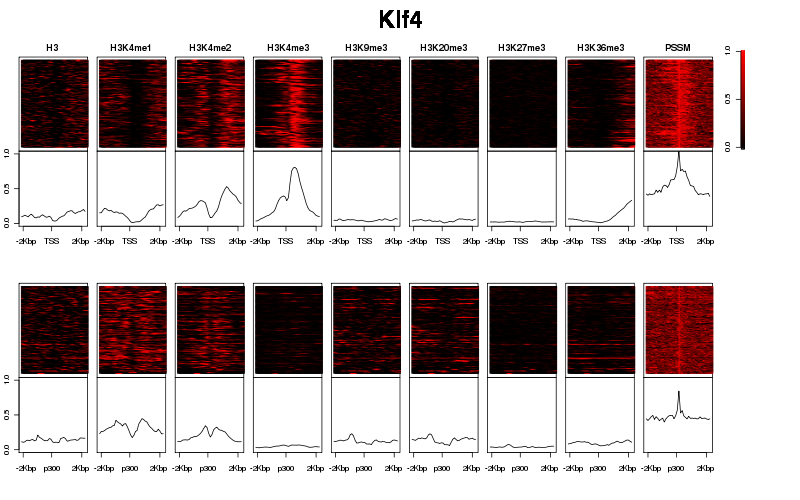

Supplement: Additional file 1 — Figures S1 and S2 [file gb-2010-11-1-r7-S1.zip › S1.Klf4.png]

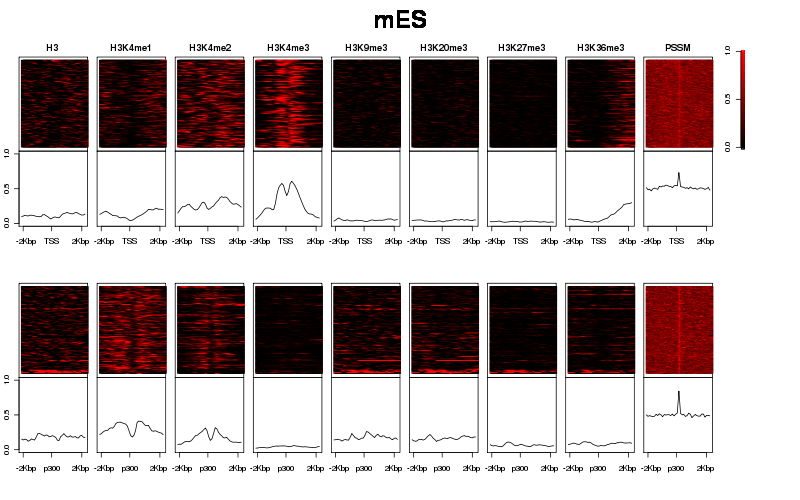

Supplement: Additional file 1 — Figures S1 and S2 [file gb-2010-11-1-r7-S1.zip › S1.mES.png]

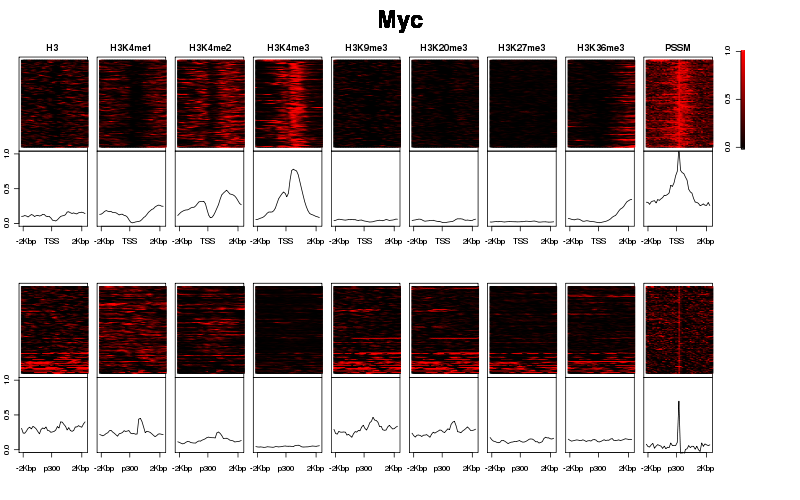

Supplement: Additional file 1 — Figures S1 and S2 [file gb-2010-11-1-r7-S1.zip › S1.Myc.png]

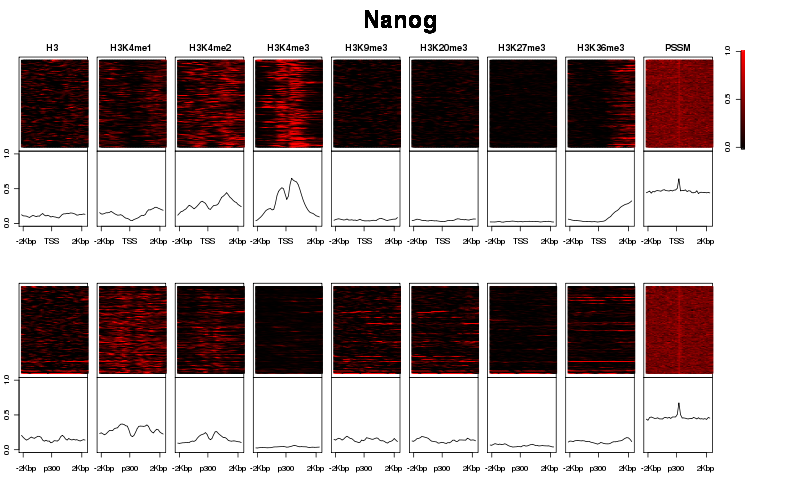

Supplement: Additional file 1 — Figures S1 and S2 [file gb-2010-11-1-r7-S1.zip › S1.Nanog.png]

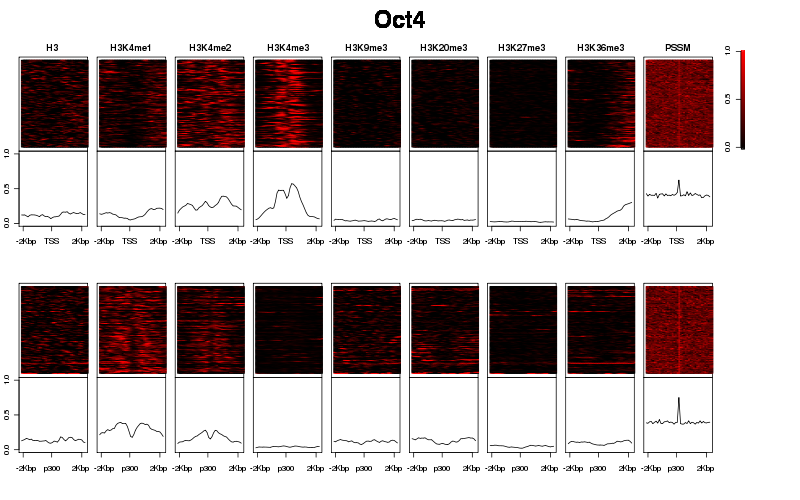

Supplement: Additional file 1 — Figures S1 and S2 [file gb-2010-11-1-r7-S1.zip › S1.Oct4.png]

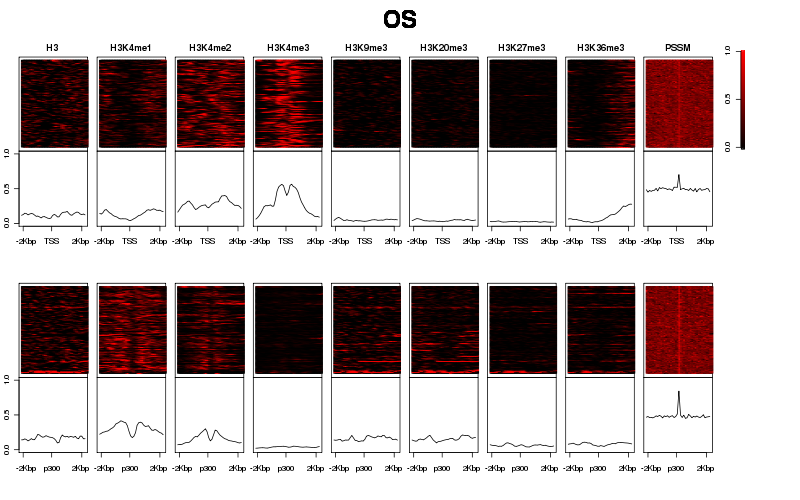

Supplement: Additional file 1 — Figures S1 and S2 [file gb-2010-11-1-r7-S1.zip › S1.OS.png]

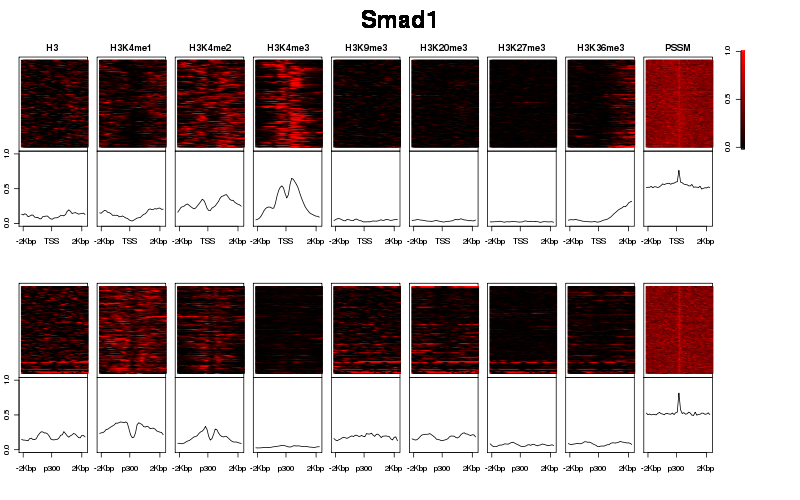

Supplement: Additional file 1 — Figures S1 and S2 [file gb-2010-11-1-r7-S1.zip › S1.Smad.png]

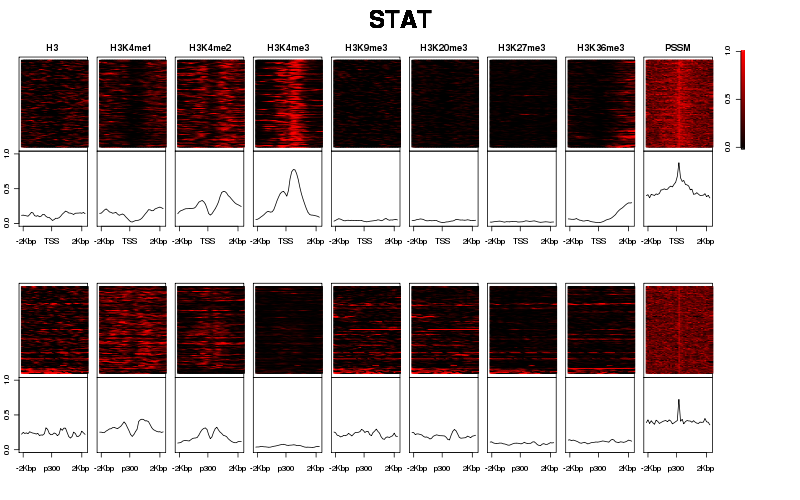

Supplement: Additional file 1 — Figures S1 and S2 [file gb-2010-11-1-r7-S1.zip › S1.STAT.png]

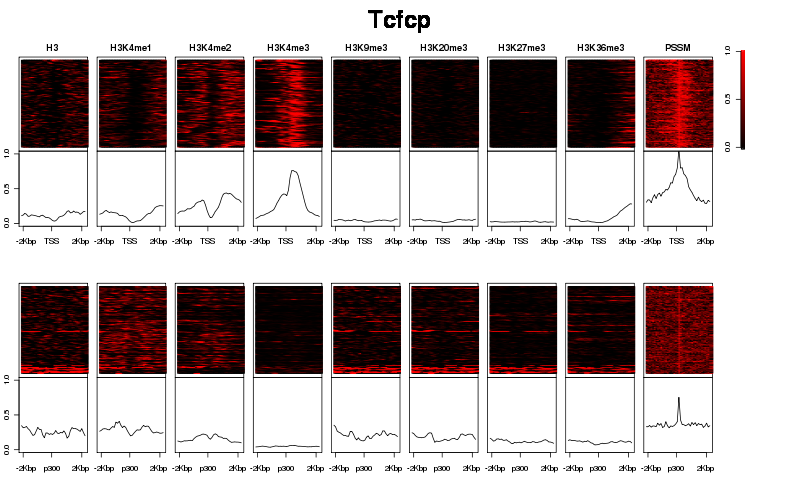

Supplement: Additional file 1 — Figures S1 and S2 [file gb-2010-11-1-r7-S1.zip › S1.Tcfcp.png]

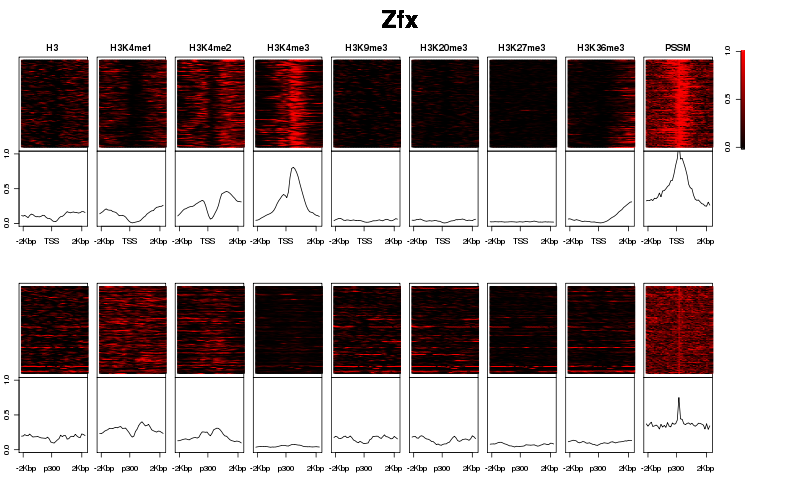

Supplement: Additional file 1 — Figures S1 and S2 [file gb-2010-11-1-r7-S1.zip › S1.Zfx.png]

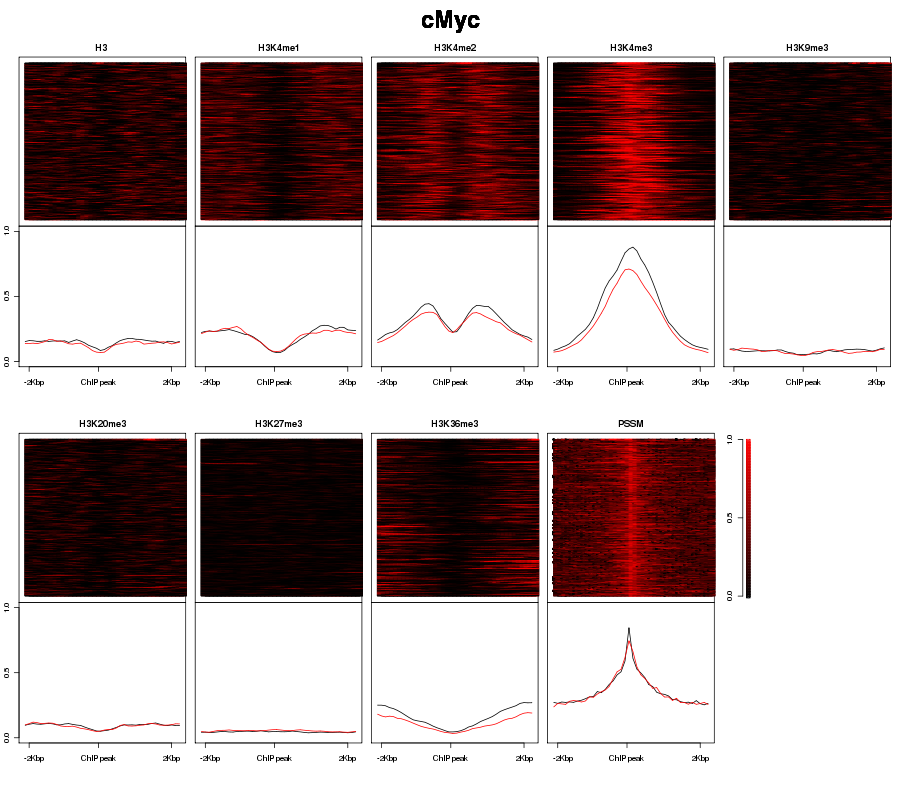

Supplement: Additional file 1 — Figures S1 and S2 [file gb-2010-11-1-r7-S1.zip › S2.cMyc.png]

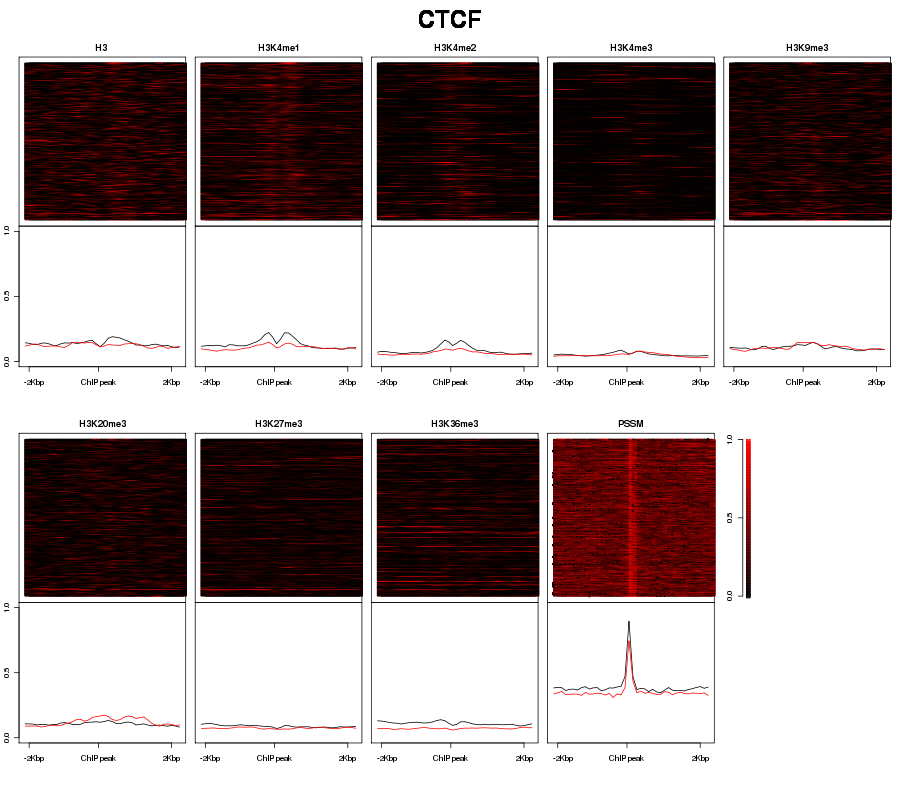

Supplement: Additional file 1 — Figures S1 and S2 [file gb-2010-11-1-r7-S1.zip › S2.CTCF.png]

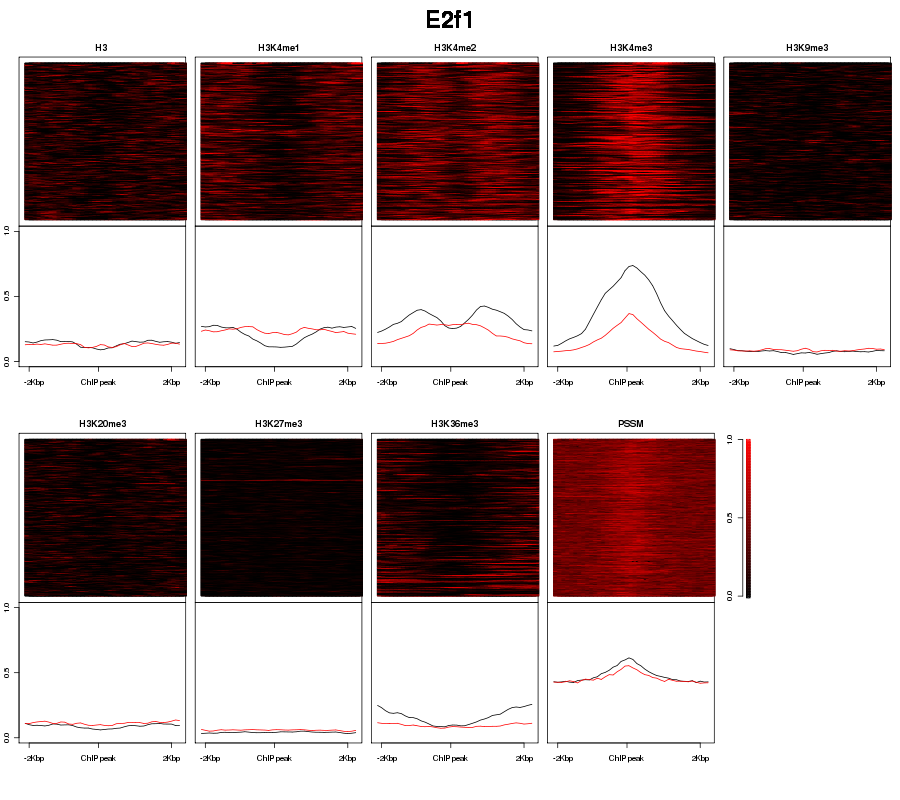

Supplement: Additional file 1 — Figures S1 and S2 [file gb-2010-11-1-r7-S1.zip › S2.E2f1.png]

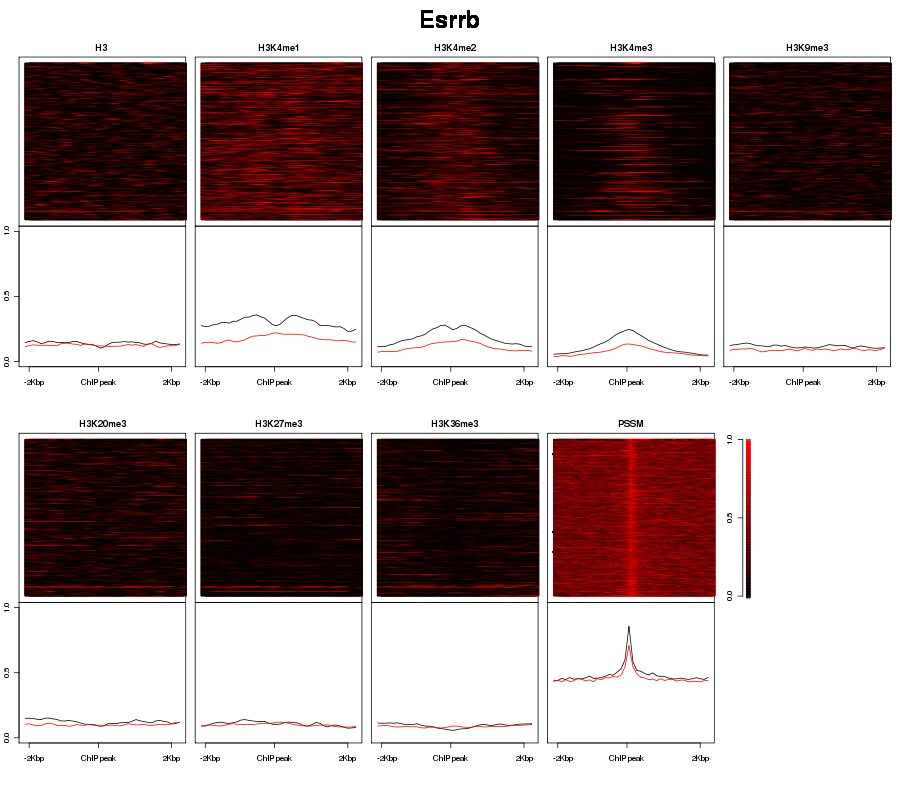

Supplement: Additional file 1 — Figures S1 and S2 [file gb-2010-11-1-r7-S1.zip › S2.Esrrb.png]

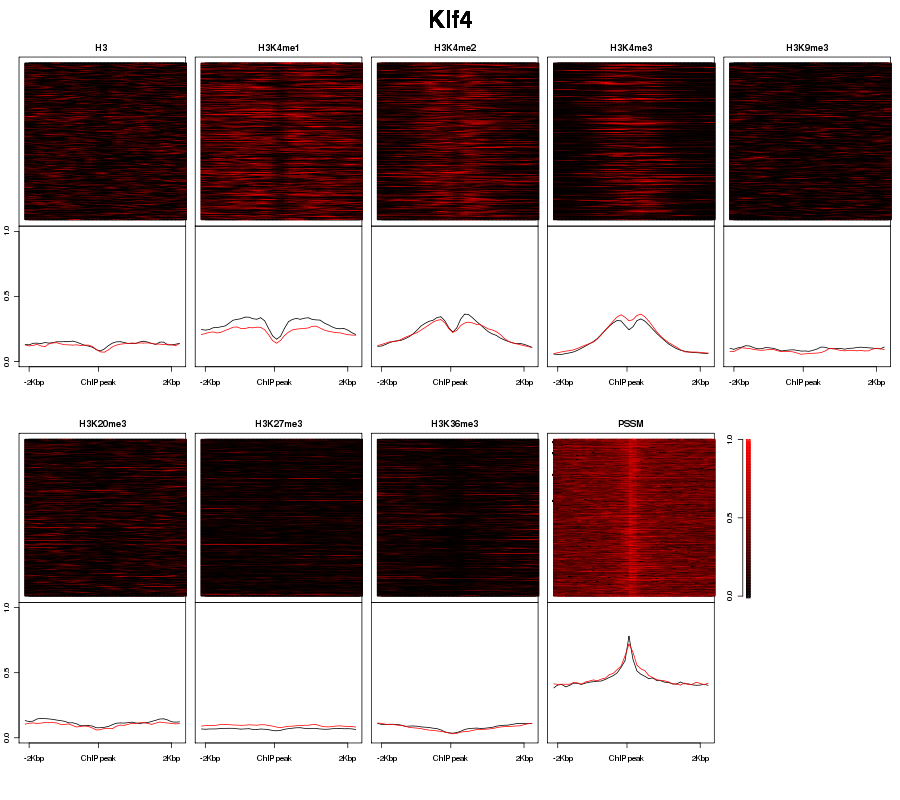

Supplement: Additional file 1 — Figures S1 and S2 [file gb-2010-11-1-r7-S1.zip › S2.Klf4.png]

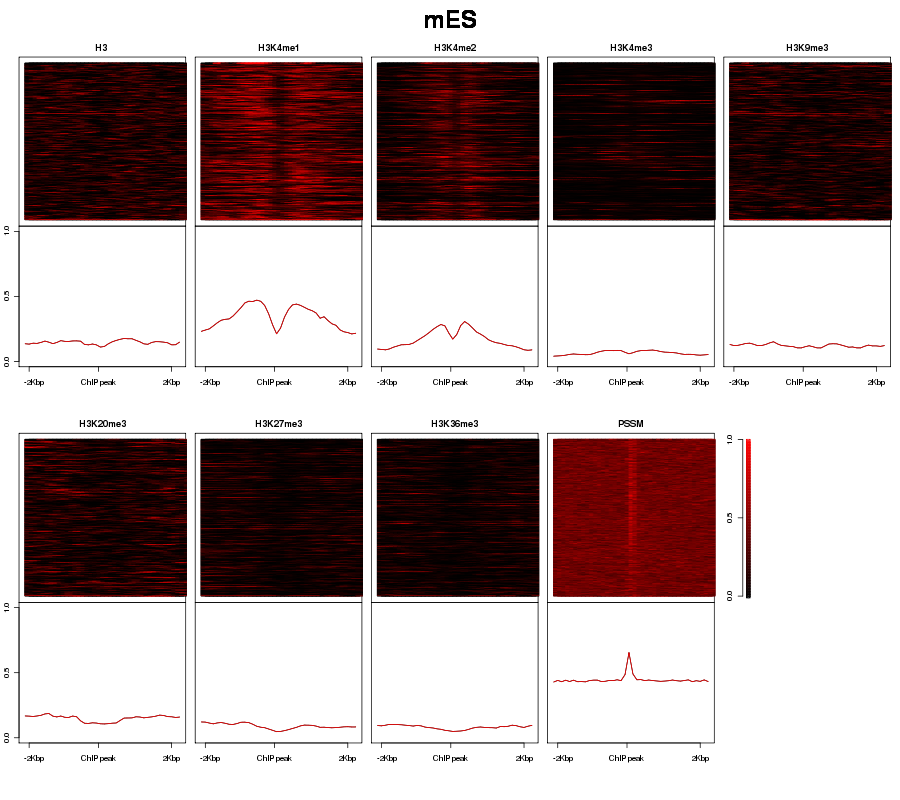

Supplement: Additional file 1 — Figures S1 and S2 [file gb-2010-11-1-r7-S1.zip › S2.mES.png]

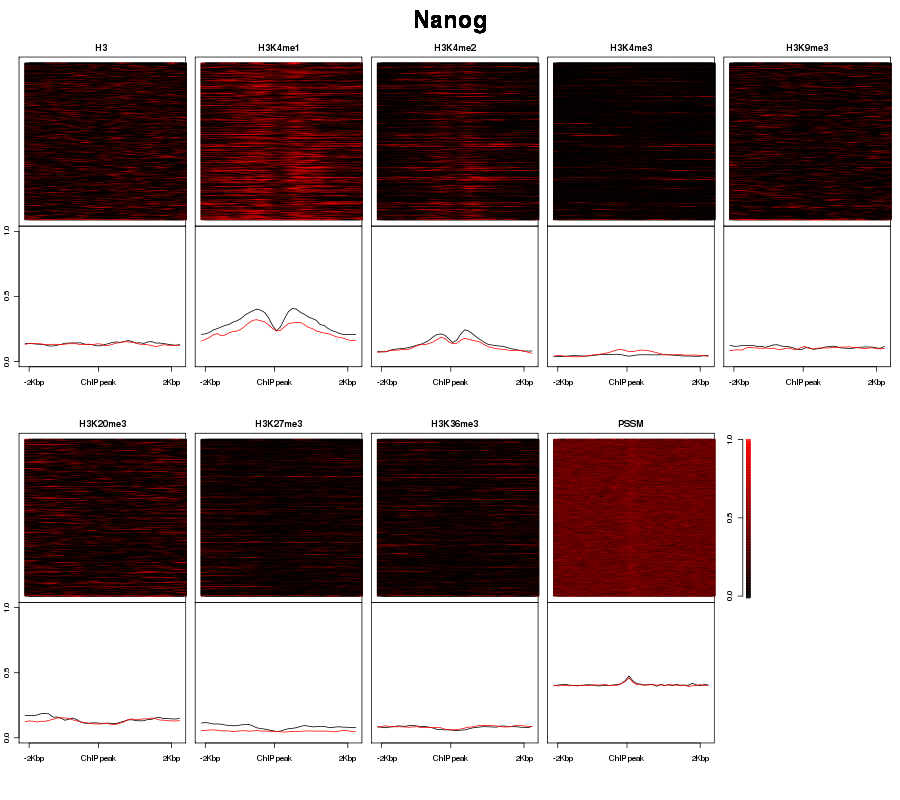

Supplement: Additional file 1 — Figures S1 and S2 [file gb-2010-11-1-r7-S1.zip › S2.Nanog.png]

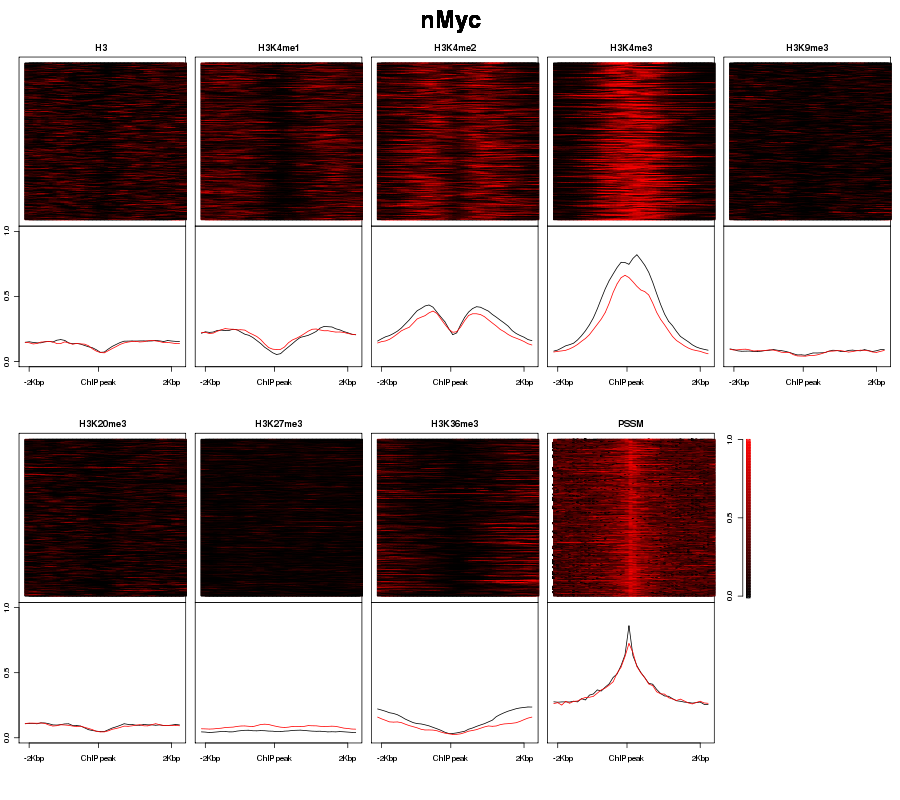

Supplement: Additional file 1 — Figures S1 and S2 [file gb-2010-11-1-r7-S1.zip › S2.nMyc.png]

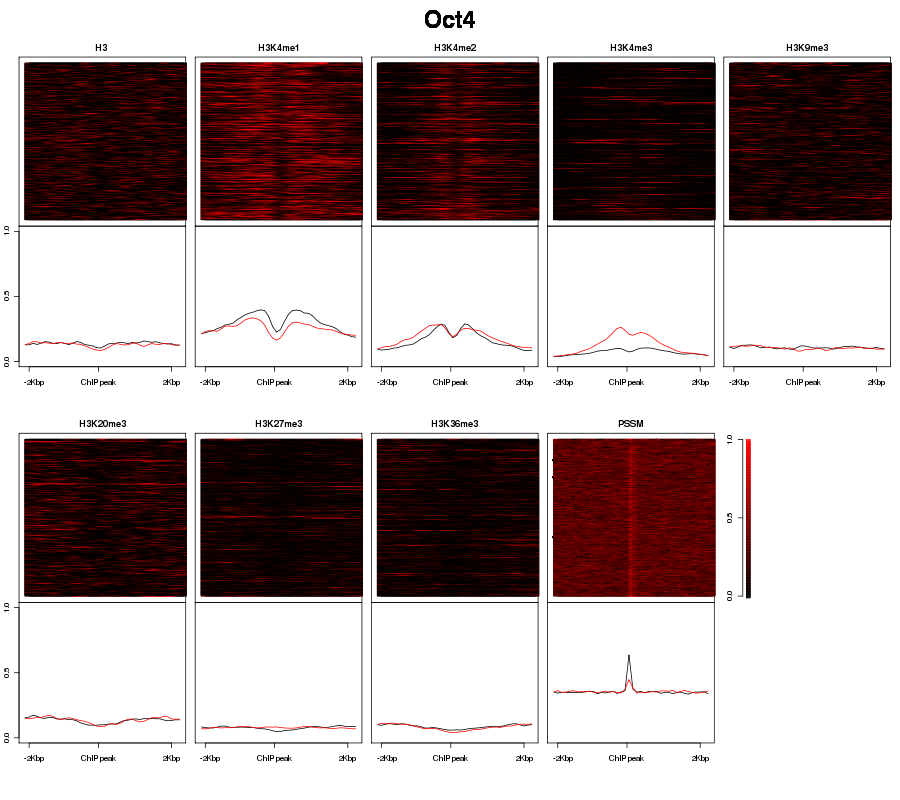

Supplement: Additional file 1 — Figures S1 and S2 [file gb-2010-11-1-r7-S1.zip › S2.Oct4.png]

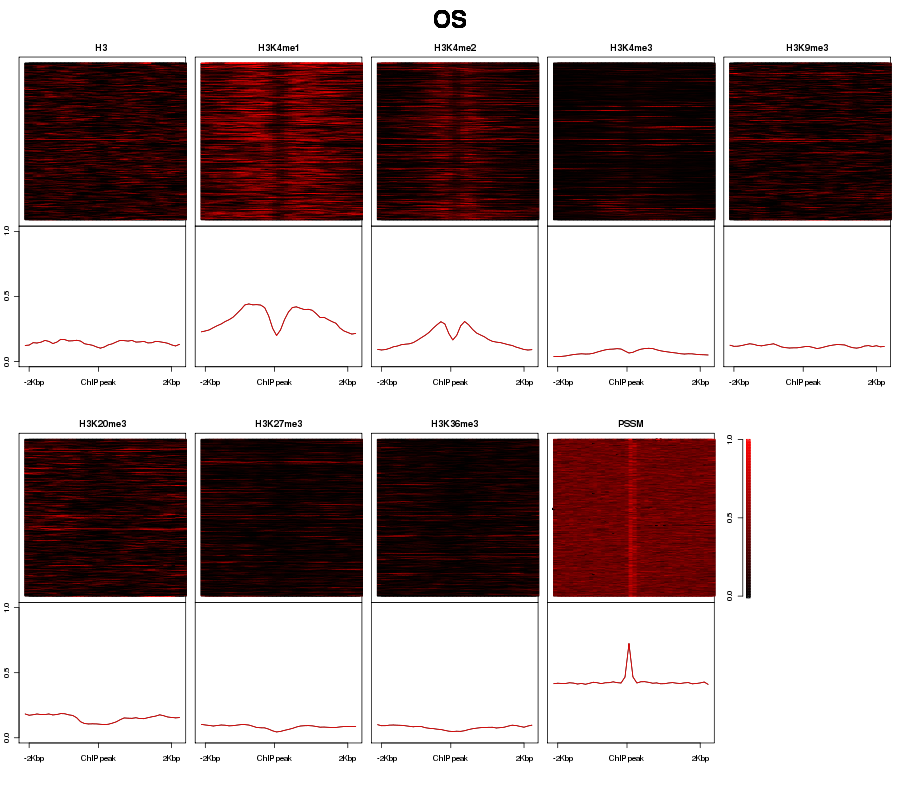

Supplement: Additional file 1 — Figures S1 and S2 [file gb-2010-11-1-r7-S1.zip › S2.OS.png]

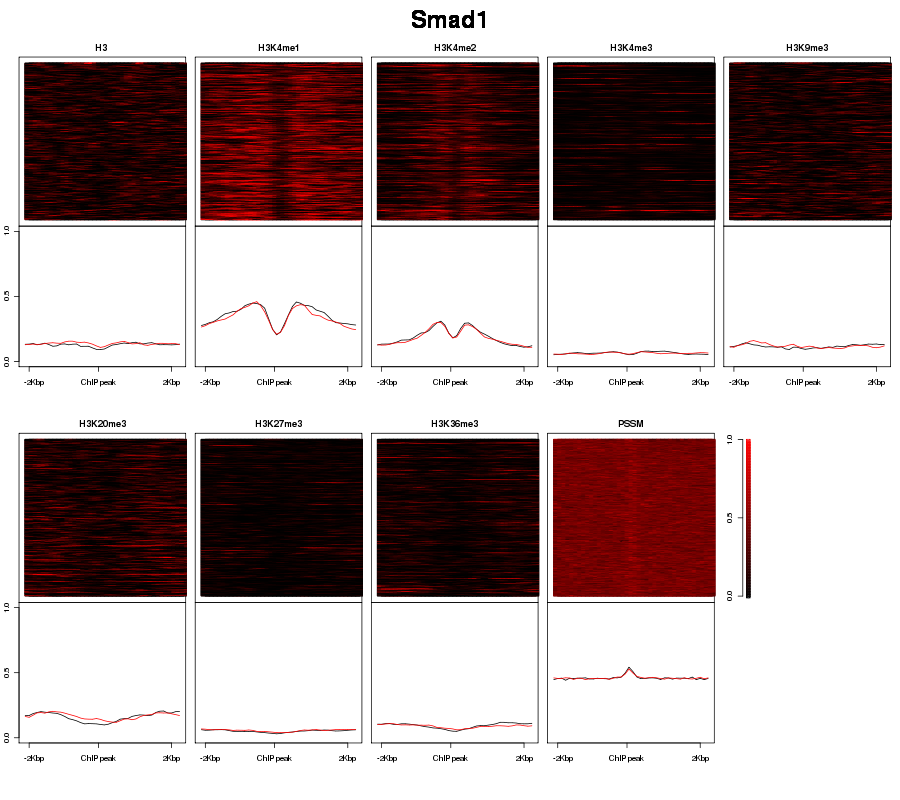

Supplement: Additional file 1 — Figures S1 and S2 [file gb-2010-11-1-r7-S1.zip › S2.Smad.png]

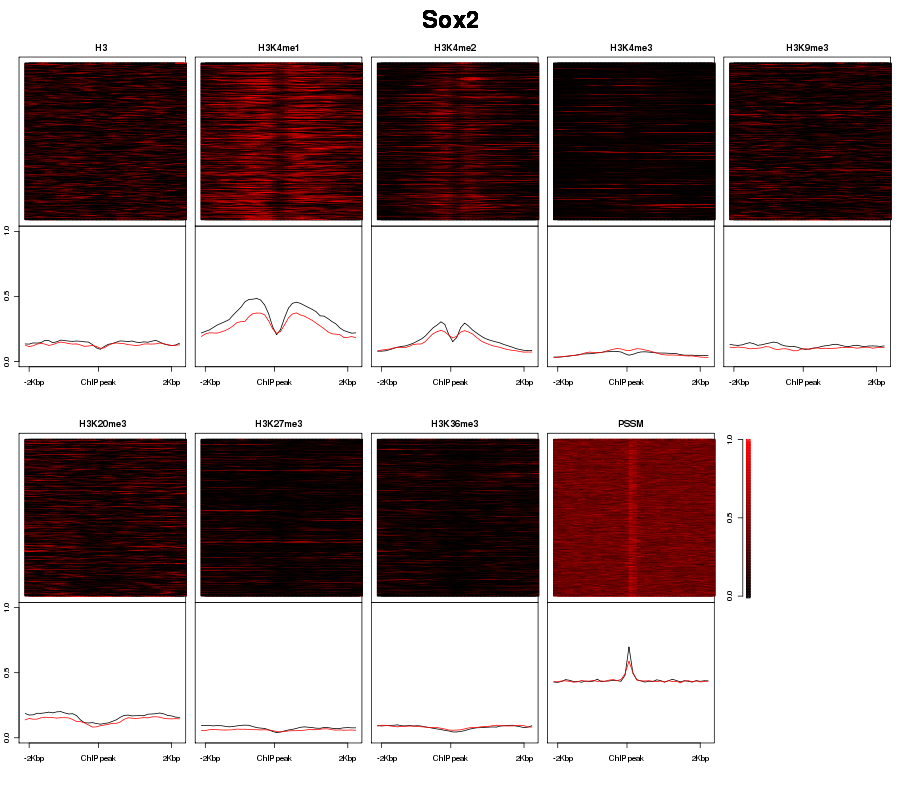

Supplement: Additional file 1 — Figures S1 and S2 [file gb-2010-11-1-r7-S1.zip › S2.Sox2.png]

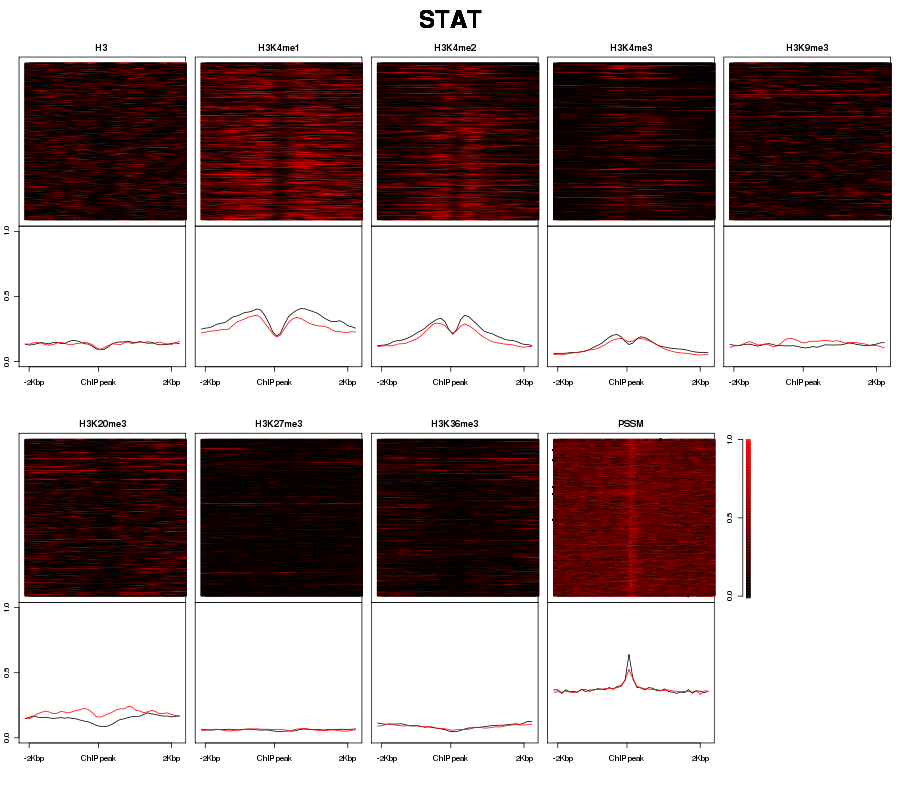

Supplement: Additional file 1 — Figures S1 and S2 [file gb-2010-11-1-r7-S1.zip › S2.STAT.png]

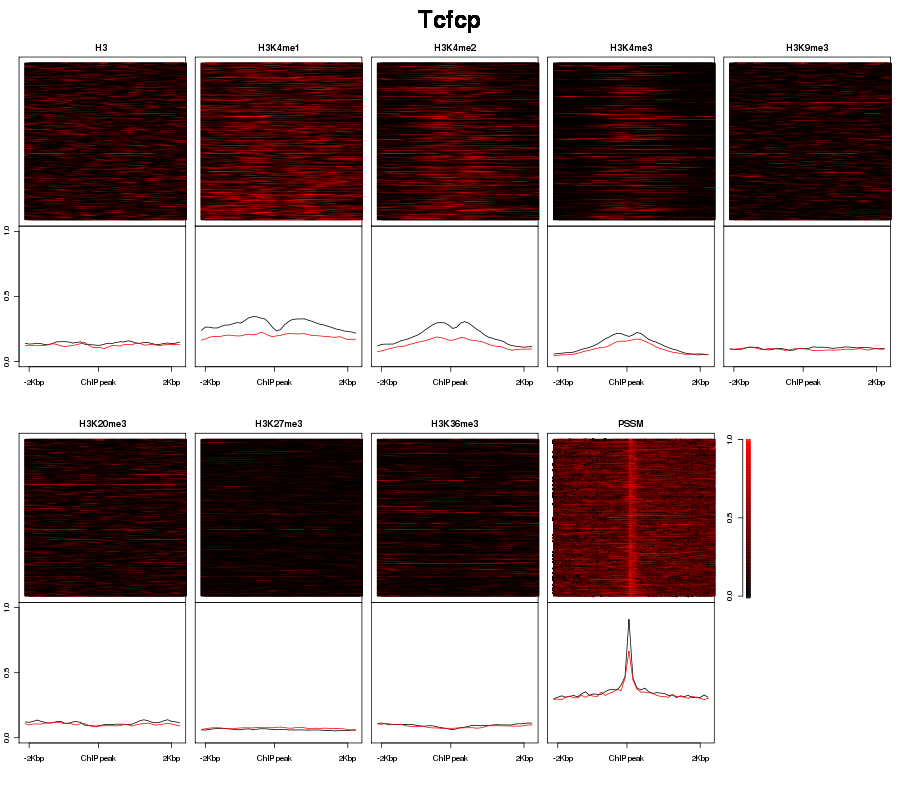

Supplement: Additional file 1 — Figures S1 and S2 [file gb-2010-11-1-r7-S1.zip › S2.Tcfcp.png]

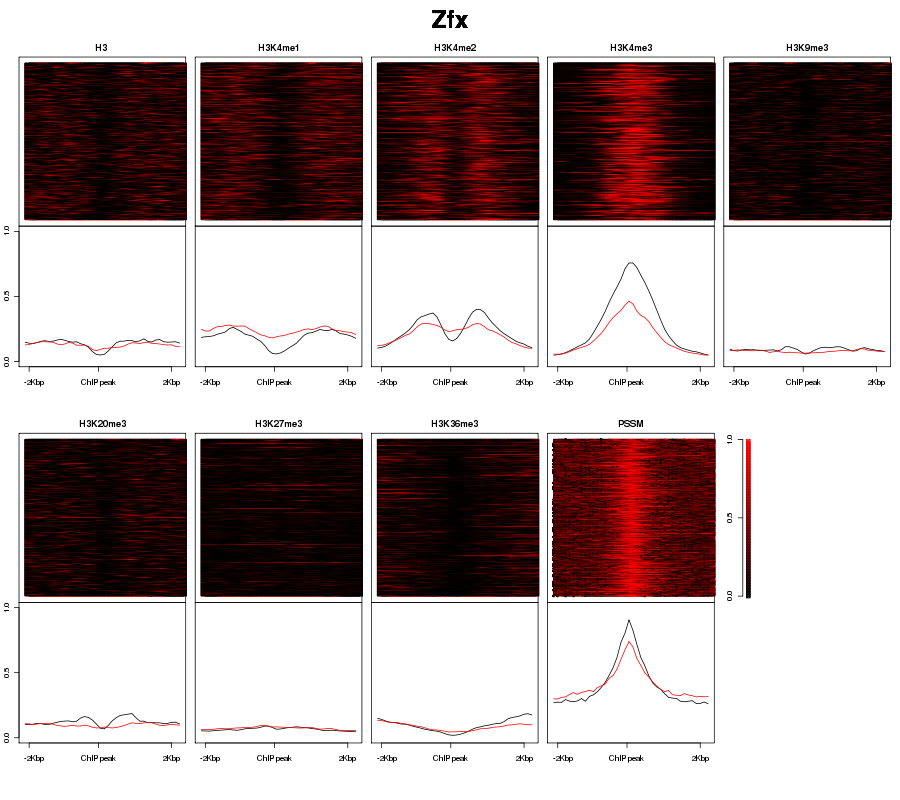

Supplement: Additional file 1 — Figures S1 and S2 [file gb-2010-11-1-r7-S1.zip › S2.Zfx.png]
