# Supplementary material for: Genome-wide prediction of transcription factor binding sites using an integrated model
Source: Genome Biol. 2010 Jan 22;11(1):R7. doi: 10.1186/gb-2010-11-1-r7 (PMC2847719; doi:10.1186/gb-2010-11-1-r7)
Supplement: Additional file 2 — Supplementary text, Figures S3 to S10, and Tables S1 to S11 [file gb-2010-11-1-r7-S2.doc]

**Additional assessment of the genome-wide TFBS predictions**

We further investigated the performance of Chromia when we changed the number of predictions. In the promoter predictions, the PPVs almost always decreased along with the decrease of the cutoff (increase of the prediction number). In contrast, the PPVs of the enhancer predictions reached a peak first and then decreased (Additional file 2, Figure S6).

**Chromia captures the temporal patterns**

To illustrate that our HMM model with a left-right structure could capture signals with temporal patterns, we generated 3 signals changing in time with distinct mean and variance (Additional file 2, Figure S10a). For the signals of the histone marks and binding motif scores, the x-axis in Additional file 2, Figure S10 is genomic position instead of time.

Additional file 2, Figure S10 shows the signals and the distribution of emission probabilities of the first and the second state in the trained HMM. The emission probabilities are represented with a mixture of 3 Gaussian distribution. The mean and variance of the Gaussians well captured the characteristics of the input signals: the first state captured the mean and the variance of the signal before *time*=12, and the second state after that time.

**
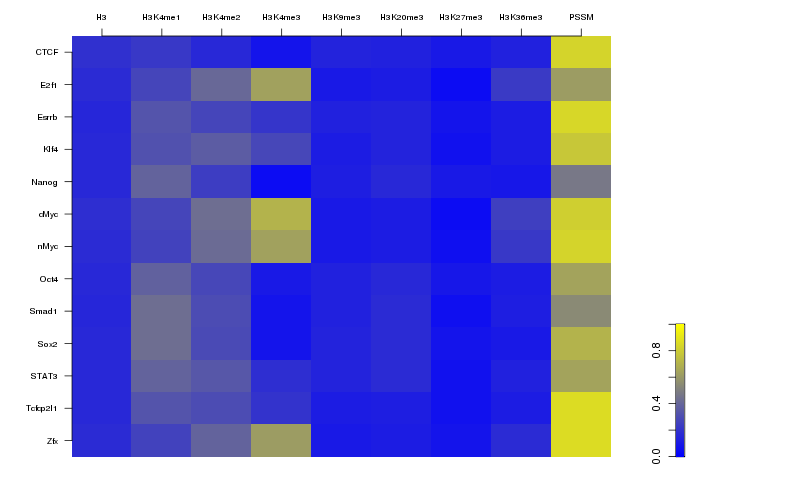
**

**Figure S3.** Histone modification intensity and PSSM score of the 13 TFs. This heatmap shows the maximum bin value in the lower panel of Figure 2. Strong H3K3me3 signals were observed associated with E2f1, cMyc, nMyc and Zfx.


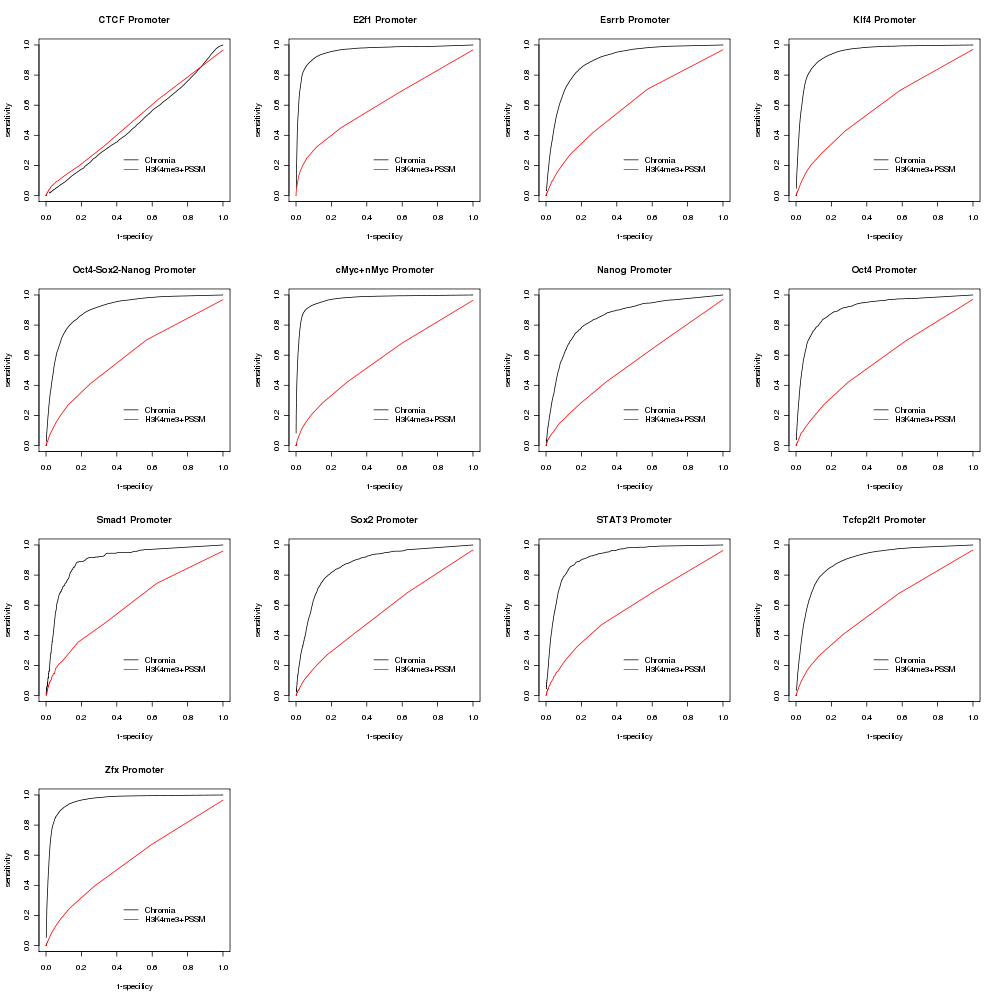


**Figure S4.** ROC curves for Chromia and the baseline method on promoter predictions in the leave-one-chromosome-out cross validations.

**
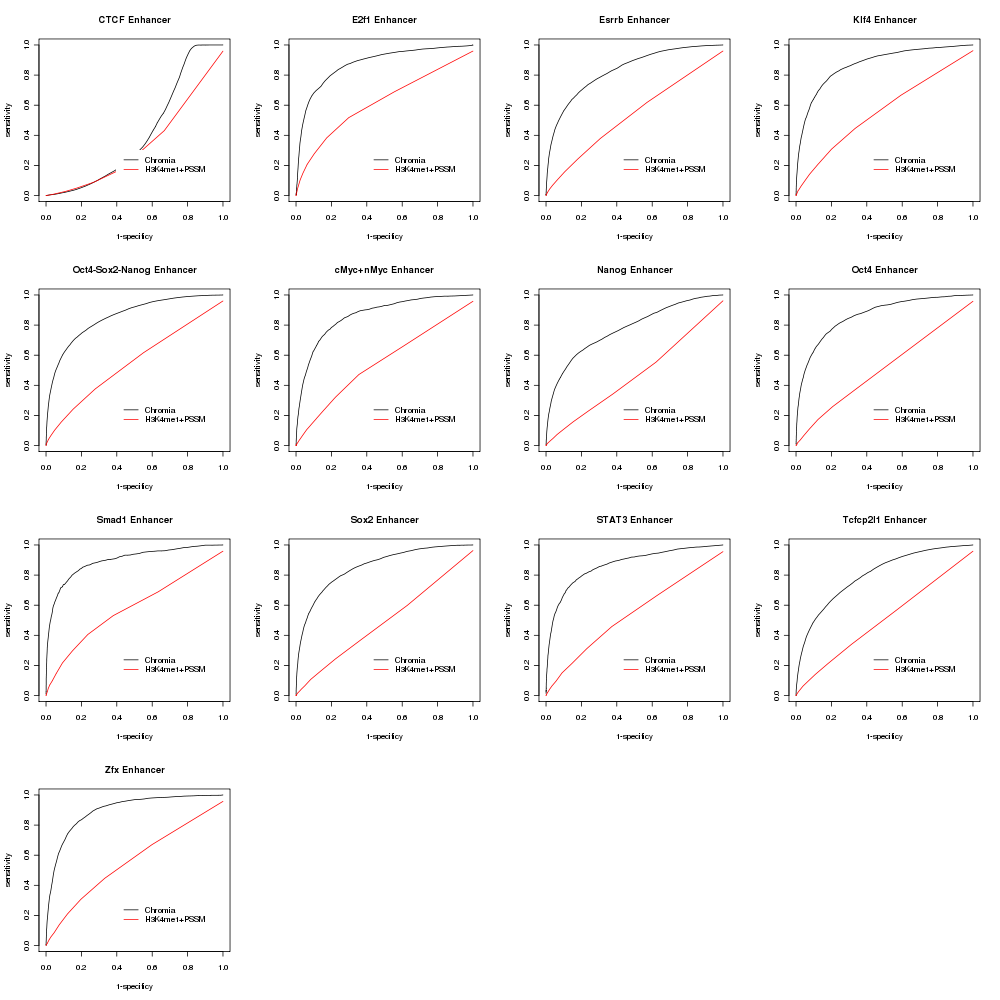
**

**Figure S5.** ROC curves for Chromia and the baseline method on enhancer predictions in the leave-one-chromosome-out cross validations.


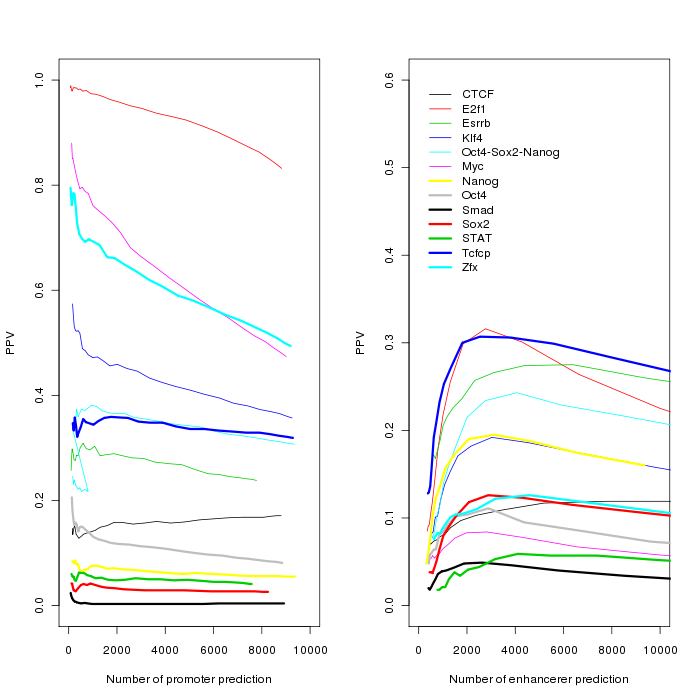


**Figure S6**. PPVs of Chromia on the 13 TFs when decreasing the log-odd score cutoff (increasing the number of predictions).

**
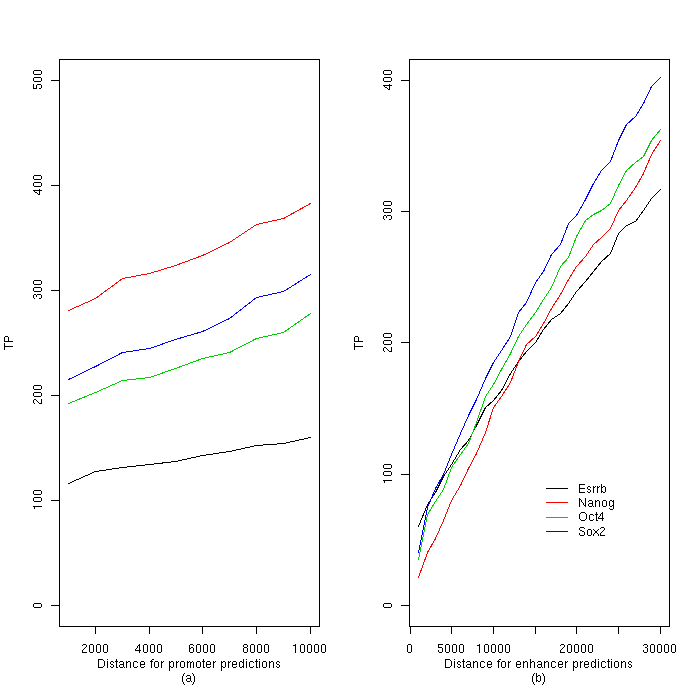
**

**Figure S7.** Number of correctly identified RNAi experiments affected genes versus the number of total predictions.


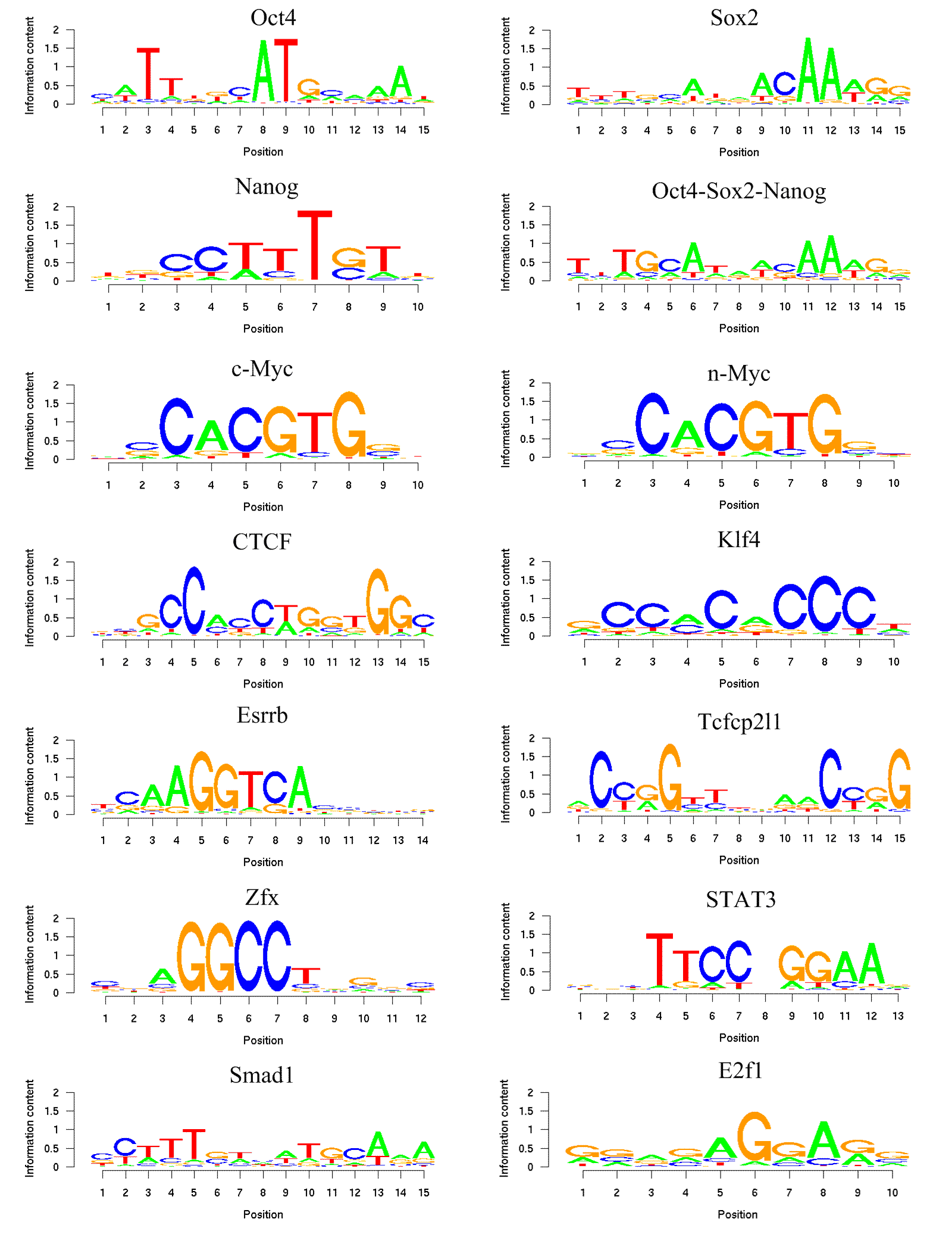


**Figure S8.** The sequence logos of the binding motifs

**
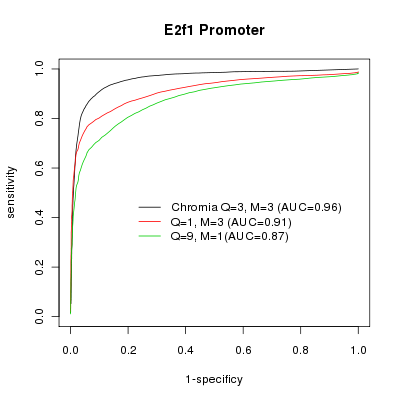
**

**Figure S9**. Testing Chromia on various parameters. We tested this on E2f1 promoter predictions (the dataset of Figure S4) in the leave-one-chromosome-out cross validations.

**
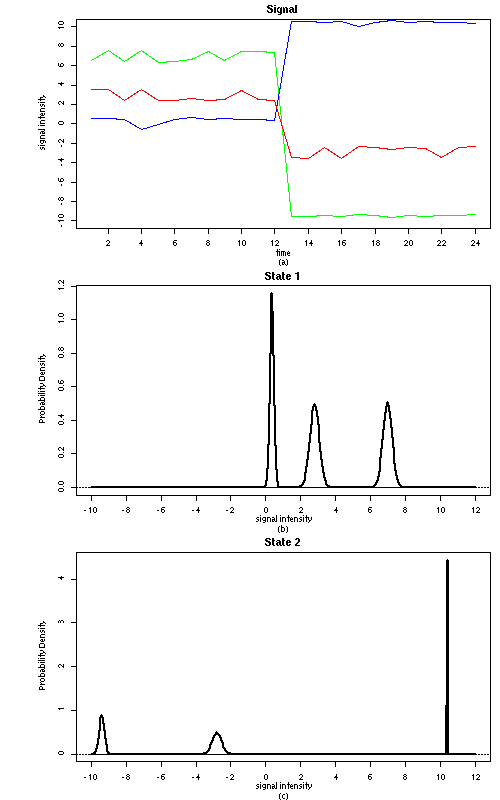
**

**Figure S10**. The input signal (a) and the probability density of the 2-state HMM trained on the signal (b and c).

**Table S**1. Location and motif enrichment of the ChIP-seq binding peaks of the 13 TFs in the mES cells.

| # of TF binding peaks | Total | Promoter^a^  ^(Promoter/Total)^ | Enhancer^b^ | Motif occurrence^c^ | | Motif ratio^d^ |
| --- | --- | --- | --- | --- | --- | --- |
|  |  |  |  | in prompters | in enhancers |  |
| CTCF | 39609 | 10629 (26.8%) | 88 (0.2%) | 7680 (72.3%) | 57 (64.8%) | 1.34 |
| E2f1 | 20699 | 12932 (62.5%) | 282 (1.4%) | 10115 (78.2%) | 95 (33.7%) | 1.34 |
| Esrrb | 21647 | 7738 (35.7%) | 409 (1.9%) | 5775 (74.6%) | 199 (48.7%) | 1.08 |
| Klf4 | 10875 | 5896 (54.2%) | 274 (2.5%) | 5114 (86.7%) | 140 (51.1%) | 1.24 |
| Nanog | 10343 | 2142 (20.7%) | 444 (4.3%) | 572 (26.7%) | 122 (27.5%) | 0.95 |
| c-Myc | 3422 | 2789 (81.5%) | 22 (0.6%) | 2467 (88.5%) | 8 (36.4%) | 1.56 |
| n-Myc | 7182 | 5552 (77.3%) | 60 (0.8%) | 4881 (87.9%) | 33 (55.0%) | 1.56 |
| Oct4 | 3761 | 1437 (38.2%) | 265 (7.0%) | 358 (24.9%) | 75 (28.3%) | 0.83 |
| Smad1 | 1126 | 246 (21.8%) | 241 (21.4%) | 74 (30.1%) | 75 (31.1%) | 0.94 |
| STAT3 | 2546 | 894 (35.1%) | 171 (6.7%) | 601 (67.2%) | 71 (41.5%) | 1.21 |
| Sox2 | 4526 | 1074 (23.7%) | 351 (7.5%) | 446 (41.5%) | 138 (39.4%) | 0.87 |
| Tcfcp2l1 | 26910 | 9680 (36.0%) | 323 (1.2%) | 7200 (74.4%) | 130 (40.2%) | 1.42 |
| Zfx | 10338 | 6811 (65.9%) | 82 (0.8%) | 5861 (86.1%) | 35 (42.7%) | 1.57 |

^a^Within 2.5kbp of a RefSeq TSS. ^b^Within 500bp of a p300 binding peak that is distal (>2.5kb) from any annotated TSS. ^c^A motif occurrence was called only if the PSSM score of a bin within 1000bp of a TF binding peaks was >99% of bins in the entire genome. Table S2 shows motif occurrence percentage of the total number of binding peaks when using 500bp and 1000bp distance cutoff from a TF binding peak. ^d^Motif ratio is defined as the ratio between the average motif score in promoters and that in enhancers.

**Table S2. Confident ChIP-seq binding peaks that contain the motifs of the 13 TFs in the mES cells.**

| # of TF binding peaks | Total | Motif occurrence | | |
| --- | --- | --- | --- | --- |
|  |  | | within 500bp^a^ | within 1000bp^b^ |
| CTCF | 39609 | | 23459 (59.2%) | 25883 (65.3%) |
| E2f1 | 20699 | | 11443 (55.3%) | 13123 (63.4%) |
| Esrrb | 21647 | | 12801 (59.1%) | 14342 (66.3%) |
| Klf4 | 10875 | | 7870 (72.4%) | 8415 (77.4%) |
| Nanog | 10343 | | 1779 (17.2%) | 2635 (25.5%) |
| c-Myc | 3422 | | 2701 (78.9%) | 2853 (83.4%) |
| n-Myc | 7182 | | 5566 (77.5%) | 5859 (81.6%) |
| Oct4 | 3761 | | 927 (24.6%) | 1159 (30.8%) |
| Smad1 | 1126 | | 249 (22.1%) | 325 (28.9%) |
| Sox2 | 4526 | | 1578 (34.9%) | 1869 (41.3%) |
| STAT3 | 2546 | | 1078 (42.3%) | 1309 (51.4%) |
| Tcfcp2l1 | 26910 | | 15022 (55.8%) | 16607 (61.7%) |
| Zfx | 10338 | | 7189 (69.5%) | 7706 (74.5%) |

A motif was called only if the PSSM score of a bin within either ^a^500bp or ^b^1000bp of a TF binding peaks was >99% of bins in the entire genome

**Table S3. Kendall’s rank correlation coefficients and corresponding p-values between the histone marks and the binding strength of the 13 TFs**

|  | H3 | H3K4me1 | H3K4me2 | H3K4me3 | H3K9me3 | H3K20me3 | H3K27me3 | H3K36me3 | PSSM |
| --- | --- | --- | --- | --- | --- | --- | --- | --- | --- |
| CTCF  (coefficient / p-value) | 0.04 | 0.123 | 0.122 | 0.125 | 0.03 | 0.046 | 0.11 | 0.089 | 0.15 |
|  | 6.4e-02 | **<2.2e-16** | **<2.2e-16** | **<2.2e-16** | 1.8e-01 | 3.4e-02 | 3.6e-07 | 3.9e-05 | **<2.2e-16** |
| E2f1  (coefficient / p-value) | 0.069 | -0.017 | 0.202 | 0.291 | -0.046 | -0.076 | -0.057 | 0.242 | 0.111 |
|  | 2.0e-03 | 4.3e-01 | **<2.2e-16** | **<2.2e-16** | 3.7e-02 | 5.0e-04 | 9.0e-03 | **<2.2e-16** | **2.3E-07** |
| Esrrb  (coefficient / p-value) | 0.015 | 0.197 | 0.165 | 0.175 | 0.033 | 0.018 | -0.025 | 0.087 | 0.152 |
|  | 5.3e-01 | **<2.2e-16** | **<2.2e-16** | **<2.2e-16** | 1.5e-01 | 4.2e-01 | 2.6e-01 | 9.8e-05 | **<2.2e-16** |
| Klf4  (coefficient / p-value) | 0.046 | 0.096 | 0.034 | 0.016 | 0.064 | 0.034 | -0.028 | 0.036 | -0.042 |
|  | 3.3e-02 | **8.0e-06** | 1.1e-01 | 4.6e-01 | 3.0e-03 | 1.2e-01 | 1.8e-01 | 9.6e-02 | 5.0E-02 |
| Nanog  (coefficient / p-value) | 0.009 | 0.123 | 0.092 | 0.069 | 0.02 | 0.004 | 0.157 | -0.01 | 0.032 |
|  | 6.6e-01 | **<2.2e-16** | 1.5e-05 | 1.0e-03 | 3.6e-01 | 8.4e-01 | **<2.2e-16** | 6.4e-01 | 1.3e-01 |
| cMyc  (coefficient / p-value) | 0.067 | 0.034 | 0.084 | 0.146 | 0.045 | 0.007 | -0.056 | 0.153 | 0.054 |
|  | 3.0e-03 | 1.2e-01 | 1.2e-04 | **<2.2e-16** | 4.5e-02 | 7.5e-01 | 1.1e-02 | **<2.2e-16** | 1.9e-02 |
| nMyc  (coefficient / p-value) | 0.026 | 0.018 | 0.118 | 0.176 | 0.02 | 0.02 | -0.09 | 0.173 | 0.054 |
|  | 2.3e-01 | 3.8e-01 | **<2.2e-16** | **<2.2e-16** | 3.4e-01 | 3.6e-01 | 2.8e-05 | **<2.2e-16** | 1.4e-02 |
| Oct4  (coefficient / p-value) | 0.014 | 0.073 | -0.04 | -0.086 | 0.026 | -0.02 | 0.06 | -0.048 | 0.101 |
|  | 5.1e-01 | 1.0e-03 | 6.1e-02 | 6.6-e5 | 2.3e-01 | 3.5e-01 | 6.0e-03 | 2.8e-02 | **3.6E-06** |
| Smad1  (coefficient / p-value) | -0.043 | 0.06 | 0.038 | 0.014 | 0.025 | 0.017 | -0.002 | 0.03 | 0.002 |
|  | 4.7e-02 | 4.0e-03 | 7.3e-02 | 4.9e-01 | 2.5e-01 | 4.2e-01 | 9.1e-01 | 1.7e-01 | 9.2e-01 |
| Sox2  (coefficient / p-value) | 0.046 | 0.108 | 0.078 | 0.034 | 0.035 | 0.02 | 0.054 | 0.017 | 0.121 |
|  | 3.9e-02 | **7.1e-07** | 1.0e-04 | 1.2e-01 | 1.1e-01 | 3.5e-01 | 1.4e-02 | 4.4e-01 | **<2.22e-16** |
| STAT3  (coefficient / p-value) | 0.02 | 0.108 | 0.099 | 0.077 | -0.003 | 0.001 | -0.038 | 0.014 | 0.052 |
|  | 3.6e-01 | **7.1e-07** | 4.4e-06 | 3.6E-04 | 8.9e-01 | 9.5e-01 | 8.1e-02 | 5.1e-01 | 1.5e-02 |
| Tcfcp2l1  (coefficient / p-value) | 0.004 | 0.184 | 0.178 | 0.145 | 0.025 | 0.053 | 0.023 | 0.053 | 0.186 |
|  | 8.4e-01 | **<2.2e-16** | **<2.2e-16** | **<2.2e-16** | 2.4e-01 | 1.4e-02 | 2.8e-01 | 1.2e-02 | **<2.22e-16** |
| Zfx  (coefficient / p-value) | 0.036 | -0.074 | 0.089 | 0.19 | 0.003 | -0.021 | -0.062 | 0.031 | 0.18 |
|  | 9.3e-02 | 1.0e-04 | 2.8e-05 | **<2.2e-16** | 8.6e-01 | 3.2e-01 | 3.0e-03 | 1.4e-01 | **<2.22e-16** |

**Table S4. Assessment of TFBS predictions using leave-one-chromosome-out** cross-validation

|  | AUC for E2f1 | AUC for Oct4-Sox2-Nanog | Running time on the 50 regions |
| --- | --- | --- | --- |
| Chromia | 0.97 | 0.81 | 7.94 sec |
| Chromia + Phastcon | 0.92 | 0.73 | 8.01 sec |
| MCAST | 0.39 | 0.42 | 0.10 sec |
| Stubb (single) | 0.49 | 0.50 | 6.78 sec |
| Stubb (multiple) | 0.42 | 0.42 | 108.32 sec |
| EEL | 0.42 | 0.53 | 12.77 sec |
| Cluster-Buster | 0.52 | 0.55 | 0.50 sec |

The end of the ROC curve was extended to (1,1) to calculate the AUC

**Table S5. Comparison between Chromia and the baseline method using leave-one-chromosome-out cross validation**.

| # of TF binding peaks | Total | Promoter | Enhancer | AUC (Promoter / Enhancer) | |
| --- | --- | --- | --- | --- | --- |
|  |  |  |  | Chromia | Baseline method |
| CTCF | 39609 | 10629 | 28980 | 0.47 / 0.41 | 0.50 / 0.34 |
| E2f1 | 20699 | 12932 | 7767 | 0.96 / 0.87 | 0.61 / 0.62 |
| Esrrb | 21647 | 7738 | 13909 | 0.89 / 0.83 | 0.60 / 0.53 |
| Klf4 | 10875 | 5896 | 4979 | 0.94 / 0.87 | 0.59 / 0.56 |
| Nanog | 10343 | 2142 | 8201 | 0.85 / 0.78 | 0.54 / 0.46 |
| c-Myc + n-Myc | 10604 | 8341 | 2263 | 0.97 / 0.87 | 0.58 / 0.55 |
| Oct4 | 3761 | 1437 | 2324 | 0.90 / 0.86 | 0.57 / 0.51 |
| Oct4-Sox2-Nanog | 18630 | 4653 | 13977 | 0.90 / 0.85 | 0.60 / 0.55 |
| Smad1 | 1126 | 246 | 880 | 0.90 / 0.89 | 0.60 / 0.58 |
| STAT3 | 2546 | 894 | 1652 | 0.92 / 0.87 | 0.58 / 0.54 |
| Sox2 | 4526 | 1074 | 3452 | 0.87 / 0.85 | 0.55 / 0.49 |
| Tcfcp2l1 | 26910 | 9680 | 17230 | 0.89 / 0.80 | 0.58 / 0.50 |
| Zfx | 10338 | 6811 | 3752 | 0.96 / 0.89 | 0.57 / 0.56 |

**Table S6. Assessment of the genome-wide TFBS predictions when |W|=1000bp. The numbers of predictions for promoters and for enhancers are 2000.**

| TF | Promoter^a^ | | | Enhancer^a^ | | |
| --- | --- | --- | --- | --- | --- | --- |
|  | Chromia | Chromia with TRANSFAC PSSM^c^ | Chromia with Phastcon Score | Chromia | Chromia with TRANSFAC PSSM | Chromia with Phastcon Score |
| CTCF | 319 (16.0%) | N/A | 287 (14.3%) | 195  (9.8%) | N/A | 222 (11.1%) |
| E2f1 | 1920 (96.0%) | 1923 (96.2%) | 1736  (86.8%) | 618 (30.9%) | 449 (22.4%) | 677 (33.9%) |
| Esrrb | 585 (29.2%) | N/A | 509 (25.4%) | 491 (24.6%) | N/A | 440 (22.0%) |
| Klf4 | 917 (45.9%) | N/A | 763 (38.1%) | 351 (17.5%) | N/A | 417 (20.8%) |
| Nanog | 138  (6.9%) | 137  (6.9%) | 118  (5.9%) | 376  (18.8%) | 302 (15.1%) | 321 (16.1%) |
| Myc^e^  (nMyc+cMyc) | 1436 (71.8%) | 1425 (71.2%) | 1232 (61.6%) | 167  (8.3%) | 153 (7.6%) | 261 (13.1%) |
| Oct4 | 240  (12.0%) | 235  (11.8%) | 199  (10.0%) | 208  (10.4%) | 206  (10.3%) | 160  (8.0) |
| Oct4-Sox2-Nanog | 384 (19.2%) | 370 (18.5%) | 344 (17.2%) | 431 (21.6%) | 432 (21.6%) | 343 (17.2%) |
| Smad1 | 6  (0.3%) | 4  (0.2%) | 11  (0.5%) | 95  (4.8%) | 109  (5.5%) | 60  (3.0%) |
| Sox2 | 63  (3.1%) | 77  (3.9%) | 76  (3.8%) | 235  (11.8%) | 305  (15.2%) | 185  (9.2%) |
| STAT3 | 99  (5.0%) | 96  (4.8) | 90  (4.5%) | 82  (4.1%) | 161  (8.1%) | 66  (3.3%) |
| Tcfcp2l1 | 716 (35.8%) | N/A | 635 (31.8%) | 595 (29.8%) | N/A | 555 (27.8%) |
| Zfx | 1320 (66.0%) | N/A | 1190 (59.5%) | 219 (10.9%) | N/A | 434 (21.7%) |

a. A prediction was considered to be a true positive (TP) if it was within |W|=1000bp of a TF binding peak. PPV is shown in parenthesis.

**Table S7. Assessment of the genome-wide TFBS predictions when |W|=500bp. The number of predictions is 2000 for promoter and for enhancer using Chromia.**

| TF | Promoter  TP (PPV) | Enhancer  TP (PPV) |
| --- | --- | --- |
| CTCF | 153 (7.6%) | 99 (5.0%) |
| E2f1 | 1527 (76.3%) | 427 (21.3%) |
| Esrrb | 395 (19.8%) | 335 (16.8%) |
| Klf4 | 684 (34.2%) | 236 (11.8%) |
| Myc (nMyc+cMyc) | 1227 (61.4%) | 106 (5.3%) |
| Nanog | 88 (4.4%) | 266 (13.3%) |
| Oct4 | 179 (8.9%) | 143 (7.1%) |
| Oct4-Sox2-Nanog | 272 (13.6%) | 300 (15.0%) |
| Smad1 | 1 (0.1%) | 64 (3.2%) |
| Sox2 | 36 (1.8%) | 161 (8.1%) |
| STAT3 | 71 (3.5%) | 55 (2.8%) |
| Tcfcp2l1 | 491 (24.6%) | 369 (18.4%) |
| Zfx | 1066 (53.3%) | 134 (6.7%) |

**Table S8. Assessment of genome-wide TFBS predictions using the same log-odd score cutoff.**

| TF | Promoter | | | | | | Enhancer | | | | | |
| --- | --- | --- | --- | --- | --- | --- | --- | --- | --- | --- | --- | --- |
|  | cutoff = 300 | | | cutoff = 250 | | | cutoff = 300 | | | cutoff = 250 | | |
|  | TP | FP | PPV  (%) | TP | FP | PPV  (%) | TP | FP | PPV  (%) | TP | FP | PPV  (%) |
| CTCF | 664 | 3557 | 15.7 | 948 | 4777 | 16.6 | 56 | 693 | 7.5 | 100 | 1098 | 8.3 |
| E2f1 | 3401 | 229 | 93.7 | 4775 | 427 | 91.8 | 67 | 499 | 11.8 | 177 | 722 | 19.7 |
| Esrrb | 973 | 2597 | 27.3 | 1302 | 3666 | 26.2 | 168 | 722 | 18.9 | 289 | 1001 | 22.4 |
| Klf4 | 1842 | 2576 | 41.7 | 2364 | 3561 | 39.9 | 80 | 701 | 10.2 | 174 | 1004 | 14.8 |
| Myc  (nMyc+cMyc) | 2582 | 1547 | 62.5 | 3268 | 2434 | 57.3 | 42 | 698 | 5.7 | 75 | 1024 | 6.8 |
| Nanog | 256 | 3879 | 6.2 | 339 | 5285 | 6.0 | 39 | 451 | 8.0 | 100 | 678 | 12.9 |
| Oct4 | 435 | 3593 | 10.8 | 539 | 4954 | 9.8 | 46 | 670 | 6.4 | 97 | 990 | 8.9 |
| Oct4-Sox2-Nanog | 710 | 3531 | 16.7 | 924 | 4810 | 16.1 | 80 | 689 | 10.4 | 165 | 959 | 14.7 |
| Smad1 | 12 | 3694 | 0.3 | 18 | 5244 | 0.3 | 21 | 672 | 3.0 | 44 | 1009 | 4.2 |
| Sox2 | 107 | 3591 | 2.9 | 147 | 4937 | 2.9 | 38 | 704 | 5.1 | 95 | 1029 | 8.5 |
| STAT3 | 191 | 3608 | 5.0 | 253 | 4965 | 4.8 | 38 | 1212 | 3.0 | 74 | 1819 | 3.9 |
| Tcfcp2l | 1509 | 2921 | 34.1 | 1980 | 3939 | 33.5 | 120 | 505 | 19.2 | 229 | 717 | 24.2 |
| Zfx | 2359 | 1526 | 60.7 | 3172 | 2353 | 57.4 | 87 | 903 | 8.8 | 148 | 1276 | 10.4 |

**Table S9. Assessment of the genome-wide TFBS predictions when |W|=1000bp. We made prediction until FP=2000.**

| TF | Promoter | | | Enhancer | | |
| --- | --- | --- | --- | --- | --- | --- |
|  | TP | PPV | AUC_2000_ | TP | PPV | AUC_2000_ |
| CTCF | 374 | 0.16 | 0.005 | 225 | 0.10 | 0.002 |
| E2f1^a^ | 7470 | 0.80 | 0.281 | 924 | 0.32 | 0.018 |
| Esrrb | 792 | 0.28 | 0.019 | 705 | 0.26 | 0.014 |
| Klf4 | 1501 | 0.43 | 0.075 | 460 | 0.19 | 0.017 |
| Nanog | 150 | 0.070 | 0.007 | 485 | 0.20 | 0.020 |
| Myc (nMyc+cMyc) | 2964 | 0.60 | 0.176 | 179 | 0.08 | 0.007 |
| Oct4 | 259 | 0.11 | 0.038 | 256 | 0.11 | 0.029 |
| Oct4-Sox2-Nanog | 454 | 0.19 | 0.013 | 603 | 0.23 | 0.012 |
| Smad1 | 6 | 0.003 | 0.003 | 103 | 0.05 | 0.038 |
| Sox2 | 64 | 0.03 | 0.008 | 277 | 0.12 | 0.024 |
| STAT3 | 86 | 0.05 | 0.021 | 103 | 0.04 | 0.013 |
| Tcfcp2l1 | 1068 | 0.35 | 0.020 | 885 | 0.31 | 0.015 |
| Zfx | 2821 | 0.59 | 0.159 | 239 | 0.11 | 0.011 |

Normalized AUC was calculated up to the FP=2000. Under this condition random guess has an AUC of 0.0004. To normalize we divided AUC with 1-specificity when FP=2000.

^a^E2f1 binding predictions in promoters has a maximum FP=1847.

**Table S10. Comparison of several computational methods on predicting TFBSs in the 20 region of sequences.** Normalized AUC was calculated up to the FP=400.

|  | Chromia | Cluster-Buster | EEL | MCAST | Stubb  (Single) | Stubb  (multiple) |
| --- | --- | --- | --- | --- | --- | --- |
| CTCF | 0.004 | 0.020 | 0.002 | **0.027** | 0.000 | 0.001 |
| E2f1 | **0.229** | 0.000 | 0.000 | 0.000 | 0.006 | 0.004 |
| Esrrb | **0.020** | 0.003 | 0.001 | 0.015 | 0.008 | 0.005 |
| Klf4 | **0.071** | 0.001 | 0.001 | 0.025 | 0.055 | 0.011 |
| Nanog | **0.008** | 0.000 | 0.000 | 0.001 | 0.000 | 0.000 |
| Myc | **0.171** | 0.001 | 0.001 | 0.006 | 0.041 | 0.034 |
| Oct4-.. | **0.043** | 0.011 | 0.000 | 0.003 | 0.000 | 0.000 |
| Oct4-Sox2-Nanog | **0.017** | 0.003 | 0.000 | 0.003 | 0.000 | 0.000 |
| Smad1 | **0.010** | 0.009 | 0.000 | 0.005 | 0.000 | 0.000 |
| Sox2 | **0.010** | 0.008 | 0.000 | 0.007 | 0.000 | 0.000 |
| STAT3 | **0.029** | 0.001 | 0.000 | 0.010 | 0.003 | 0.003 |
| Tcfcp | **0.025** | 0.004 | 0.000 | 0.019 | 0.001 | 0.002 |
| Zfx | **0.133** | 0.000 | 0.001 | 0.060 | 0.124 | 0.052 |

To normalize we divided AUC with 1-specificity when FP=400.

**Table S11. 20 genomic regions used in the genome-wide comparison with other methods. Total base pairs covered is 513,846,568.**

| Chr3: 76142362 – 142405818 |
| --- |
| Chr15: 3155231 – 32702850 |
| Chr9: 90111680 -118338591 |
| chrX: 20271704 -142831400 |
| Chr12: 3247101 – 31589168 |
| Chr13: 30567516 -48483288 |
| Chr2: 113679704 -126760908 |
| Chr16 :75431264 -98119428 |
| Chr7: 86986037 -101951804 |
| Chr1: 181674757 -196922031 |
| Chr18: 32836181 -60304384 |
| Chr15: 89969566 -103433335 |
| Chr11: 16851091 -31005173 |
| Chr11: 86295357 -97137682 |
| Chr6 :133923648 -149520669 |
| Chr6 :92264626 -103288266 |
| Chr5 :3155799 -20990949 |
| Chr18: 68618824 -80143597 |
| Chr7 :101952498- 116337593 |
| Chr3 :57338175 -76052755 |
